# Clinical biomarker discovery by SWATH-MS based label-free quantitative proteomics: impact of criteria for identification of differentiators and data normalization method

**DOI:** 10.1186/s12967-019-1937-9

**Published:** 2019-05-31

**Authors:** Mythreyi Narasimhan, Sadhana Kannan, Aakash Chawade, Atanu Bhattacharjee, Rukmini Govekar

**Affiliations:** 1 Advanced Centre for Treatment, Research and Education in Cancer, Tata Memorial Centre, Kharghar, Navi Mumbai 410210 India; 2 BARC Training School Complex, Homi Bhabha National Institute, Anushakti Nagar, Mumbai, 400094 India; 3 Department of Plant Breeding, Swedish University of Agricultural Sciences, Alnarp, Sweden; 4 Section of Biostatistics, Centre for Cancer Epidemiology, Tata Memorial Centre, Kharghar, Navi Mumbai 410210 India

**Keywords:** Proteomic, LC–MS, SWATH, Normalization, p-value, Fold change

## Abstract

**Background:**

SWATH-MS has emerged as the strategy of choice for biomarker discovery due to the proteome coverage achieved in acquisition and provision to re-interrogate the data. However, in quantitative analysis using SWATH, each sample from the comparison group is run individually in mass spectrometer and the resulting inter-run variation may influence relative quantification and identification of biomarkers. Normalization of data to diminish this variation thereby becomes an essential step in SWATH data processing. In most reported studies, data normalization methods used are those provided in instrument-based data analysis software or those used for microarray data. This study, for the first time provides an experimental evidence for selection of normalization method optimal for biomarker identification.

**Methods:**

The efficiency of 12 normalization methods to normalize SWATH-MS data was evaluated based on statistical criteria in ‘Normalyzer’—a tool which provides comparative evaluation of normalization by different methods. Further, the suitability of normalized data for biomarker discovery was assessed by evaluating the clustering efficiency of differentiators, identified from the normalized data based on p-value, fold change and both, by hierarchical clustering in Genesis software v.1.8.1.

**Results:**

Conventional statistical criteria identified VSN-G as the optimal method for normalization of SWATH data. However, differentiators identified from VSN-G normalized data failed to segregate test and control groups. We thus assessed data normalized by eleven other methods for their ability to yield differentiators which segregate the study groups. Datasets in our study demonstrated that differentiators identified based on p-value from data normalized with Loess-R stratified the study groups optimally.

**Conclusion:**

This is the first report of experimentally tested strategy for SWATH-MS data processing with an emphasis on identification of clinically relevant biomarkers. Normalization of SWATH-MS data by Loess-R method and identification of differentiators based on p-value were found to be optimal for biomarker discovery in this study. The study also demonstrates the need to base the choice of normalization method on the application of the data.

**Electronic supplementary material:**

The online version of this article (10.1186/s12967-019-1937-9) contains supplementary material, which is available to authorized users.

## Background

Liquid chromatography-mass spectrometry (LC–MS) based quantitative proteomic profiling has substantially contributed to identification of disease biomarkers for improved diagnosis/better prognostication or to monitor response to therapy [1–3]. This is achieved through assessment of the ability of differentiators, identified by quantitative proteomics, to segregate the comparison groups distinctly by cluster analysis—an essential feature of biomarkers. Success of this process depends not only on selection of appropriate clinical samples and sample processing strategies, but also on mass spectrometry based-factors such as the depth of LC–MS profile and occurrence of instrumental or non-instrumental errors in the data. Therefore, use of mass spectrometers with capabilities for in-depth profiling and data processing strategies which reduce biases, errors and optimize the desired outcome is necessary.

The recent feature in MS—Sequential window acquisition of all theoretical fragment-ion spectra (SWATH) provides in depth profiling by data independent acquisition (DIA) [4]. It is preferred for profiling clinical samples, as in data or information-dependent acquisition (IDA) data from low expressers is lost permanently [5]. SWATH not only provides for fragmentation of almost all ions but also for re-interrogation of data, after detection capabilities are improvised to identify more number of proteins [4]. These features are conducive to profiling of clinical samples which are available in amounts insufficient for enrichment and are unavailable for reanalysis. A testimony to this is the wide use of SWATH-MS in clinical proteomics after its discovery in 2012. PubMed results show that 44% (20/45) of the SWATH-MS studies on clinical samples published till date are aimed at biomarker discovery or therapeutic target identification.

However, a feature in quantification by SWATH-MS, if overlooked, can hinder biomarker identification. Unlike labelled quantification by IDA wherein all samples for relative quantification are run together, in label-free quantification by SWATH, each sample from the comparison group is run individually in MS. This increases the probability of both systematic and random error. Intervention to reduce these variations by ‘normalization’ is thus a prerequisite to subsequent analysis of SWATH data for identification of differentiators. The data from reported SWATH-MS studies is normalized using either methods provided by the MS instrument-based software or those used to normalize microarray data [6–9]. As the source of systematic bias differs between MS and microarray, it is essential to experimentally validate the appropriate normalization strategy for SWATH data.

The present study was undertaken to experimentally identify an appropriate normalization method for SWATH-MS data. The statistical tool ‘Normalyzer’, which compares the efficiency of diverse methods to normalize ‘omics’ data based on statistical criteria [10], was used to achieve the same. Fu et al. [11] in their study to identify the optimal analysis chain have reported total ion current normalization as optimal for SWATH-MS data based on statistical end-point. Further, considering the wide application of SWATH-MS in biomarker identification, in this study we have supplemented the statistical evaluation with biologically relevant criteria of precise stratification of comparison groups by cluster analysis. Towards this (a) Normalization of data was assessed using ‘Normalyzer’ to identify the optimal method of normalization based on statistical criteria (b) from the data normalized by different methods in Normalyzer, differentiators between comparison groups were identified based on p-value, fold change and combination of both. The potential of these differentiators to segregate comparison groups distinctly, was assessed by cluster analysis. Detection of optimal method for normalization of SWATH-MS data and optimum criteria for identification of differentiators would have an impact on biomarker discovery.

## Methods

**Fig. 1:**
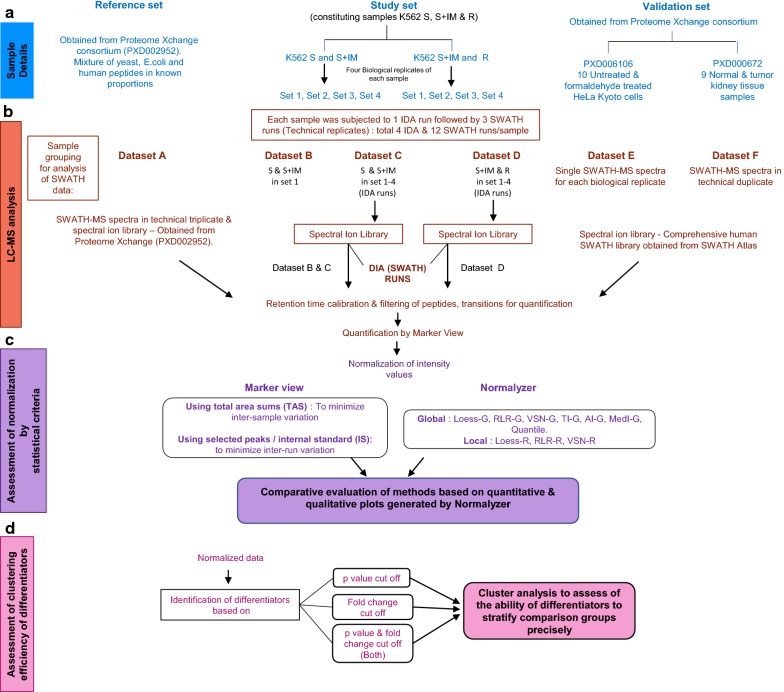
Scheme of experiments. It describes: **a** Samples used in this study which include IM-sensitive K562 cells (S) untreated or treated with imatinib (S + IM), IM-resistant K562 cells (R) and 3 datasets from public domain. **b** Generation of spectral ion library for all comparison groups in A from information dependent acquisition (IDA) data and generation of quantitative proteomic profile by data independent acquisition (DIA) using Sequential window acquisition of all theoretical fragment-ion spectra (SWATH). **c** Normalization of SWATH data using different methods. **d** Identification of differentiators based on p-value, fold change and combination of both followed by cluster analysis of the identified differentiators

The scheme of experiments employed to identify the normalization strategy optimum for SWATH-MS data is depicted in Fig. 1. It involves:

A. Inclusion of a quantitatively defined dataset from public domain, generated from hybrid of peptides from three different sources mixed in defined proportions, to serve as a ‘reference set’. Generation of datasets using K562 cells for quantitation by SWATH-MS, referred to as ‘study set’ which includes one set with smaller number of samples and two sets with larger number of samples. Further, inclusion of two datasets from public domain comprising of larger sample size, to serve as ‘validation set’ to confirm the findings in the study set.
B. SWATH-MS analysis of reference, study and validation set.
C. Normalization of SWATH-MS data obtained from reference, study and validation sets using methods in Marker view software and Normalyzer and identification of optimum method of normalization based on statistical criteria.
D. Identification of differentiators from this normalized data based on criteria of p value, fold-change and both, followed by cluster analysis of these differentiators.

### Details of samples for SWATH-MS analysis

Details of samples used for SWATH-MS analysis are summarized in Fig. 1a. The reference set was obtained from data published by Navarro et al. [12] wherein samples were prepared by mixing known proportions of constituent proteome (i.e. with known fold-difference in quantities). Samples with a hybrid of human, yeast and *E. coli* peptides referred to as HYE124 had differences in relative proportions of the constituent peptides and served as control (65% w/w human, 30% w/w yeast, 5% w/w *E. coli* peptides) and test (65% w/w human, 15% w/w yeast, 20% w/w *E. coli* peptides). SWATH runs of these samples in technical triplicate and their corresponding spectral ion library deposited in Proteome Xchange consortium (identifier-PXD002952), was used for SWATH data analysis.

A ‘study set’ was generated in our laboratory using K562, an erythroleukemic cell line (generous gift from Dr. Tadashi Nagai, Jichi Medical University, Tochigi, Japan). It was maintained in RPMI 1640 medium supplemented with 10% FBS and 1% antibiotic (Gibco, Thermo Fisher Scientific, USA). K562 harbours BCR/ABL oncogene which encodes a constitutively active tyrosine kinase, whose activity is inhibited by the small molecular inhibitor imatinib (IM). Inhibition of BCR/ABL activity by imatinib is known to cause quantitative changes in the proteome of K562 cells [13, 14].

Thus IM-sensitive K562 cells (S) untreated or treated with imatinib (S + IM) and IM-resistant K562 cells (R) were analyzed. For S cells, treatment with imatinib was carried out at 0.75 µM concentration for 12 h, a condition observed to inhibit BCR/ABL activity without compromising on cell viability (data not shown). R cells were always maintained in medium containing 0.75 µM imatinib. SWATH-MS profiles were generated for four biological replicates of S, S + IM and R, each run-in triplicate (Fig. 1a, b).

The ‘validation set’ constituted SWATH data deposited by Tan et al. [15]. and Guo et al. [16] in Proteome Xchange consortium with identifiers PXD006106 and PXD000672, respectively. SWATH runs of ten biological replicates of HeLa Kyoto cells untreated (UT) and treated with formaldehyde (FA) were obtained from PXD006106 while duplicate SWATH runs of normal (N) and tumorous (T) kidney tissue samples from nine patients were obtained from PXD000672.

### Preparation of K562 lysates for LC–MS analysis

To prepare whole cell lysate, 1 × 10^6^ cells were suspended in 100 µl SDS buffer (10% glycerol, 2% SDS, 5% β-mercaptoethanol and 62.5 mM tris pH 6.8), boiled for 10 min and centrifuged at 13,000×*g* for 15 min. The supernatant was collected and acetone precipitated with 1 ml chilled acetone to remove detergents. Protein pellet thus obtained was denatured by resuspending in 6 M urea and protein concentration was determined by Bradford assay [17]. 10 µg protein was subjected to in-solution trypsin digestion. Briefly, the denatured proteins were reduced by incubating with 200 mM dithiothreitol (DTT) for 1 h at room temperature. It was followed by alkylation with 200 mM iodoacetamide (IAA) for 1 h in dark. Before trypsin digestion, urea concentration was adjusted to 0.6 M using 1 mM CaCl_2_. In-solution digestion was carried out by adding proteomic grade trypsin (Sigma Aldrich, USA) in the ratio of 1:50 trypsin: protein (w/w) and incubated for 16 h at 37 °C. Peptides were then desalted using C18 spin columns (Pierce, Thermo Fisher Scientific, USA), dried in a speed vac and reconstituted with 0.1% Formic acid (FA) in water to get a final concentration of 0.5 µg/µl.

### LC–MS/MS data acquisition for the study set

Each sample in the study set was spiked with 1 pmol/µl of digested *Escherichia coli* β-galactosidase (β-gal) peptides (Sciex, USA), before injection, which served as internal standard. The samples were then injected into Eksigent ekspertTM nano-LC 400 with cHiPLC^®^ system, with trap column (200 µmX 0.5 mm) and analytical column (75 µmX 15 cm), both packed with 3 µl ChromXp C18 (120 Å). For reverse phase HPLC, 0.1% FA in water and 0.1% FA in acetonitrile (ACN) served as solvent A and B respectively. A gradient elution of 225 min, with increasing percentage of mobile phase B was used to elute the peptides at a flow rate of 300 nl/min. Eluate from the column was analyzed in a positive ion mode on Triple TOF 5600 + (Sciex, USA) mass spectrometer.

Each sample was subjected to 1 IDA run for spectral ion library generation followed by 3 DIA (SWATH) runs, which served as technical replicates. Thus, with four biological replicates, K562 S, S + IM and R cells had 4 IDA runs and 12 SWATH runs each (Fig. 1b). IDA mode involved a survey scan over a mass range of 350–1250 m/z and MS/MS scan over 200–1800 m/z for top 30 precursor ions with rolling collision energy, 50 mDa mass tolerance and accumulation time of 250 ms for MS and about 50 ms for MS/MS.

For DIA-SWATH acquisition, the instrument was tuned to a looped product ion mode. A sequential isolation window width of 25 m/z (with 1 m/z overlap) covering a mass range of 350–1250 m/z was set, resulting in 36 overlapping windows. The accumulation time was 50 ms for MS scan and 80 ms for MS/MS scan, thereby making a total cycle time of about 3 s. The conditions used to generate data by Navarro et al. [12]. Guo et al. [16] and that used to generate data experimentally in this study were comparable, while data generated by Tan et al. [15] used 64 variable wide precursor ion selection window. Further, samples in the reference set and validation set were spiked with indexed retention time (iRT) peptides for retention time calibration while those in the study set were spiked with *E. coli* β-gal peptides.

### Generation of spectral ion library for the study set

The reference set from Navarro et al. [12] was referred to as Dataset A. The data acquired from S, S + IM and R sets were further grouped for comparison into datasets (Fig. 1b and Table 1). Only one out of the four sets of S and S + IM each, was considered as dataset B while all four together as dataset C. All four sets of S + IM and R were included in dataset D. The validation sets from Tan et al. [15] and Guo et al. [16] were referred to as dataset E and F respectively.

**Table 1:** Details of datasets

Datasets	Source	Constituents	Purpose—in this study
Dataset A	Pride ID—PXD002952	3 samples of 65% human, 30% yeast, 5% *E. coli* peptides (Control) 3 samples of 65% human, 15% yeast, 20% *E. coli* peptides (Test)	Reference set—a well-defined dataset with predictable quantification
Dataset B	In vitro experiments carried out in this study	3 samples of K562/S cells (Control) 3 samples K562/S + IM cells (Test)	Study set to check comparability of observations in defined (A) versus undefined (B) datasets
Dataset C	In vitro experiments carried out in this study	12 samples of K562/S cells (Control) 12 samples K562/S + IM cells (Test)	Larger dataset (C) to check the application of observations from small dataset (B)
Dataset D	In vitro experiments carried out in this study	12 samples of K562/S + IM cells (Control) 12 samples K562/R cells (Test)	Larger dataset (D) to check the consistency of observations in independent large datasets
Dataset E	Pride ID—PXD006106	10 samples of untreated HeLa Kyoto cells (Control) 10 samples of formaldehyde treated HeLa Kyoto cells (Test)	Validation set to check the consistency of observations in independent large datasets
Dataset F	Pride ID—PXD000672	18 non-tumorous kidney tissue samples (Control) 18 tumorous kidney tissue samples (Test)	Validation set to check the consistency of observations in independent large datasets

A common spectral ion library was generated for datasets B and C while a separate library was created for dataset D. The spectral ion library for datasets B, C and D was generated by pooling the IDA runs of the corresponding biological replicates and analysing in Protein Pilot software v4.5 (Sciex, USA) with paragon algorithm, to obtain protein identities. The parameters used were as follows: Cysteine alkylation—IAA, digestion—trypsin and no special factor was chosen. The search effort was set to ‘thorough ID’ and false discovery rate (FDR) analysis was enabled. Proteins identified with 1% FDR were considered. The search was carried out against UniProt database (November 2016 release) containing human proteins as well as *E. coli* β-gal. The result (.group) file thus generated served as the spectral ion library. For dataset A the spectral ion library deposited by Navarro et al. [12], generated by pooling individual libraries of constituent human, yeast and *E. coli* peptides, was used. For datasets E and F comprehensive human SWATH library with about 10,000 proteins deposited in SWATH Atlas by Rosenberger et al. [18] was used.

### SWATH data analysis

Spectral alignment and targeted data extraction of the swath runs of all six datasets were carried out in Peak View 2.2 software using MS/MS ALL with SWATH acquisition microapp (Sciex, USA). Proteins from spectral ion library identified with 1% FDR were first imported into Peak View 2.2 software. Retention time calibration was carried out using iRT peptides for datasets A, E and F and *E. coli* β- gal peptides for datasets B-D. Processing settings were used to filter the ion library, where up to 6 peptides per protein and 6 transitions per peptide with peptide confidence threshold of 99% and FDR of 1%, were chosen for quantification. Modified peptides were excluded from extraction. Extracted ion chromatogram (XIC) window was set to 5 min for datasets A, B, C, E, F and 15 min for dataset D with XIC width of 50 ppm. The MS/MS extracted peak areas from the filtered results were exported to Marker View software v1.3 (Sciex, USA) for quantification. The marker view output raw data file with list of proteins and their peak areas were used for further analysis.

### Normalization of SWATH Data

The raw data of all datasets was processed and analyzed in Normalyzer (Fig. 1c), wherein it was log2 transformed and then normalized globally (G) or locally (R) using 10 statistical methods. Global normalization is carried out without consideration of affiliation of the sample such as replicate, control group, test group, etc. [10]. In SWATH-MS since each sample is run individually, errors can arise irrespective of their origin. Thus, in the present study global normalization methods were included. However, since the study focuses on identification of normalization method conducive to biomarker identification, retention of distinguishing features of the comparison groups was necessary while normalizing the data. This was achieved by including local normalization methods for analysis [10]. The normalization methods include locally estimated scatterplot smoothing (Loess-R, Loess-G) which assumes non-linear relationship between the bias in the data and magnitude of protein intensity; robust linear regression (RLR-R, RLR-G) which assumes that the bias in data is linearly dependent of the magnitude of the measured protein intensity; variance stabilization normalization (VSN-R, VSN-G) which aims at making the sample variances nondependent from their mean intensities and bringing the samples onto the same scale; quantile normalization which forces the distribution of the samples to be the same; total intensity (TI-G), average intensity (AI-G) and median intensity (MedI-G) normalization methods wherein intensity of each variable is divided by sum of intensities, mean of sum of intensities, median intensities of all variables respectively [8, 10].

Marker view v1.3 along with quantitation also provides options for sample normalization using either total area sums (TAS) wherein total area of all peaks in a sample is considered or using area of the selected peaks or internal standard (IS). In this study spiked iRT peptides and trypsin digest of *E. coli* β- gal served as an internal standard for dataset A, E, F and datasets B–D respectively. In TAS as well as IS normalization, the peak areas of each sample were normalized by multiplying with its corresponding scale factor. The scale factor for TAS method was obtained by dividing the average of total area of all samples by the total area of each sample while for IS method the average area of internal standard of all samples was divided by the area of internal standard of each sample. Data normalized by the above two methods i.e. TAS and IS was log2 transformed before running through Normalyzer, to generate the evaluation report.

The normalization efficiency of all 12 methods was assessed through ‘Normalyzer’ quantitatively by pooled intragroup coefficient of variation (PCV) and qualitatively by relative log expression (RLE) plot as reported in earlier studies [8, 10].

### Identification of differentiators from normalized data and cluster analysis

Differentiators were identified from the data of all datasets normalized by 12 methods based on the criteria of p-value, fold-change and a combination of both (Fig. 1d). To obtain p-value, log2 transformed data, normalized by different normalization methods from comparison groups were assessed by Student’s t-test using IBM SPSS statistics 21. Differences in protein intensities with p-value ≤ 0.05 were considered statistically significant and chosen as differentiators. The fold change difference in protein levels was calculated from the peak area values and a cut-off of 1.5-fold change was applied. Further, the efficiency of differentiators obtained from data normalized using the 12 methods to segregate the comparison groups was assessed by cluster analysis. The peak areas of differentiators identified using p-value (≤ 0.05), fold change (1.5 fold) and combination of both were used as inputs for cluster analysis (Fig. 1d) in Genesis software v.1.8.1. Hierarchical clustering was performed with the following parameters: Agglomeration rule − Average linkage WPGMA and Calculation parameters − Cluster experiments.

## Results

### Identification and quantitation of proteins by SWATH-MS

In this study, each of the four biological replicates of K562 S, S + IM and R, underwent one IDA run for the generation of spectral ion library followed by three DIA runs for SWATH-MS analysis, thereby resulting in a total of 4 IDA and 12 DIA runs for K562 S, S + IM and R each. Samples with improper chromatogram were eliminated from analysis sets leaving 11 runs each in S and R in datasets C and D respectively (Fig. 1b). In dataset F, there were 2 technical replicates for each sample. Upon spectral alignment and filtering of ion library, 4404, 1450, 1757, 1808, 7057 and 5316 proteins that fulfilled the criteria (described in Methods under ‘SWATH data analysis’) were further used for quantification of datasets A, B, C, D, E and F respectively. Quantities of the identified proteins were further assessed for variation.

### Assessment of variation in un-normalized data

**Fig. 2:**
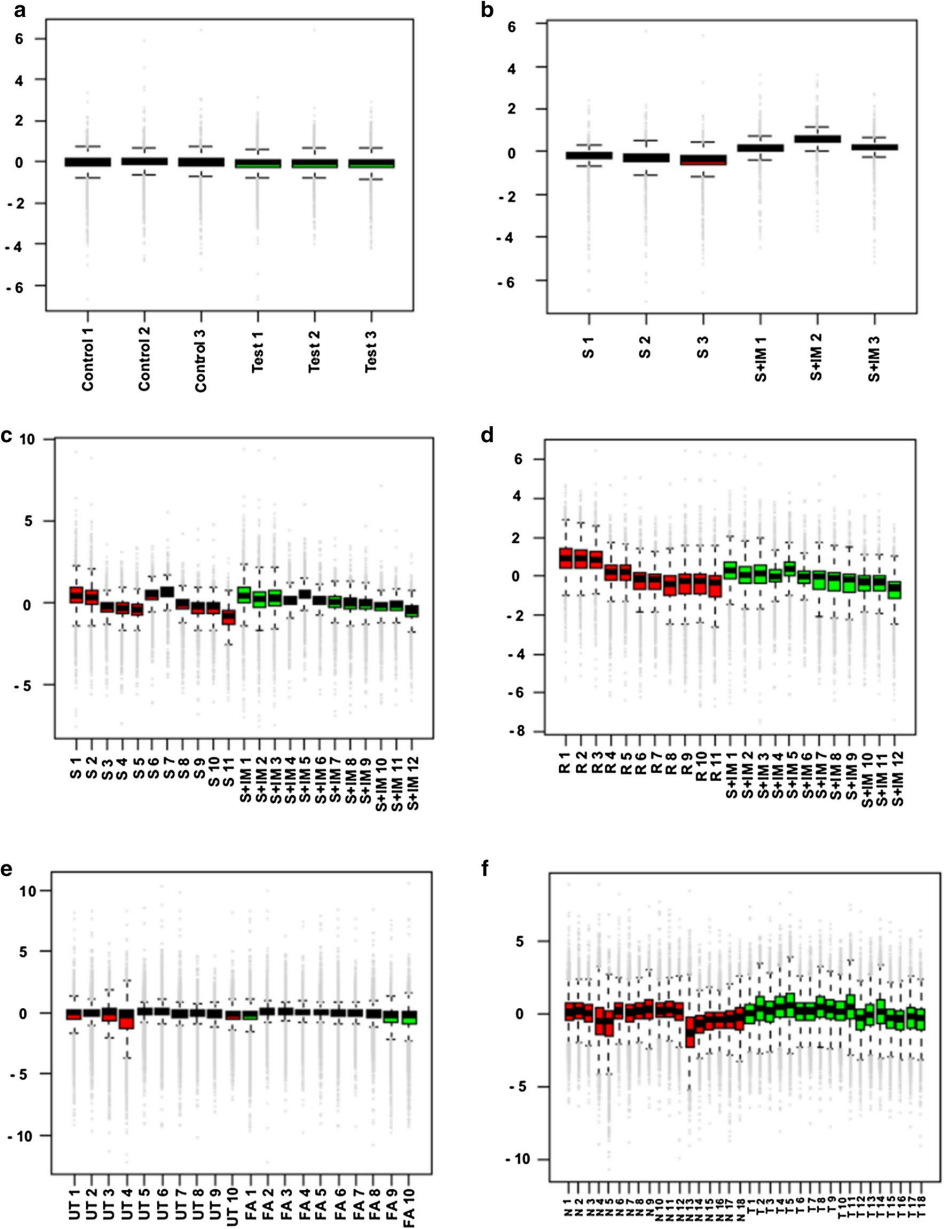
Analysis of unnormalized SWATH data for datasets A–F (**a**–**f**) by RLE plot: Qualitative assessment of the spread of data shows that the test and control groups vary in their spread of values in all datasets except **a** and **e**

The quantified log2 transformed ‘un-normalized’ data of each dataset was evaluated based on RLE plot, which assesses the inter- and intra-group alignment of the replicates qualitatively. In RLE plot, samples should be aligned around zero. Any deviation would indicate discrepancies in the data [10]. Among the datasets constituted of single set of samples, alignment around zero was seen in all the representative samples of dataset A (Fig. 2a) and 50% of those in dataset B (Fig. 2b). Datasets C (Fig. 2c), D (Fig. 2d) and F (Fig. 2f) comprising of multiple sets, showed considerable deviation from zero in replicates as well as between groups in RLE plots, indicating the need for normalization of SWATH-MS data.

### Identification of optimum method for normalization using ‘Normalyzer’

**Fig. 3:**
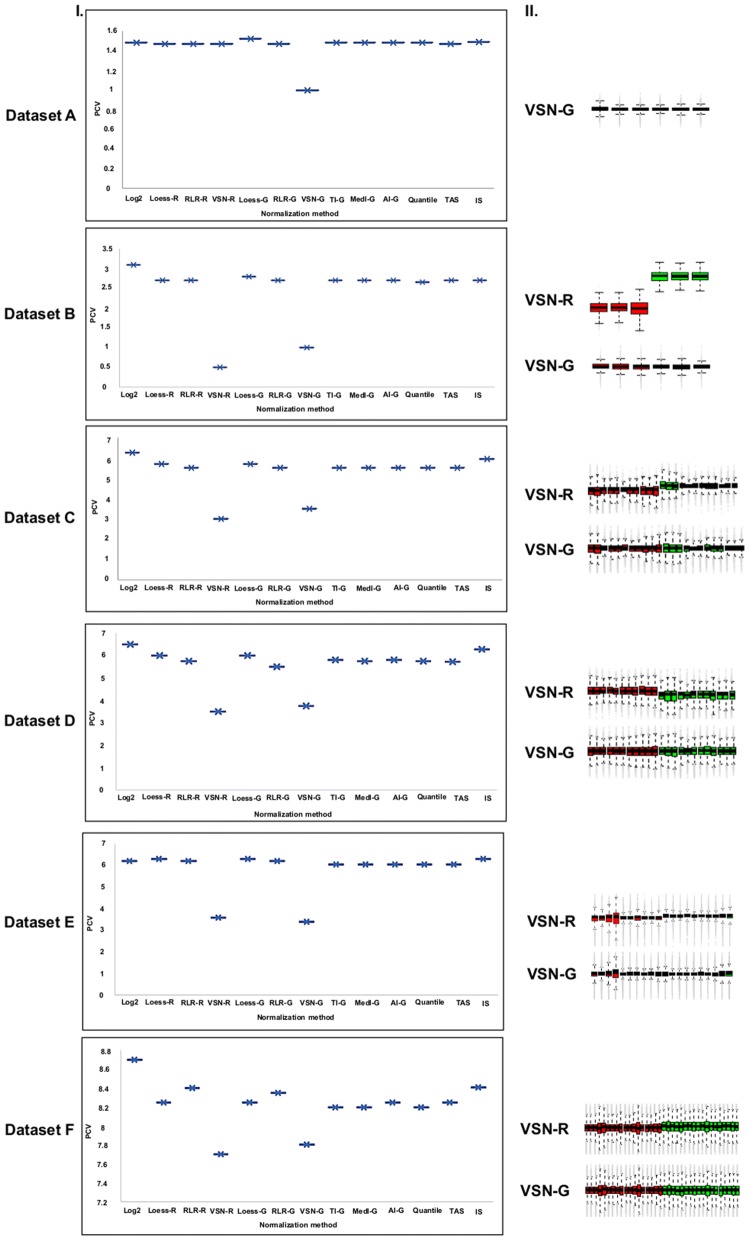
**I** PCV plot: Quantitative assessment of twelve normalization methods indicates that VSN-G has less PCV in all datasets along with VSN-R in datasets B–F. **II** RLE plot: Qualitative analysis of methods with less PCV, by RLE plot revealed good inter group alignment only in VSN-G in all datasets

The efficiency of 12 different normalization methods to normalize datasets A–F, was assessed quantitatively and qualitatively in ‘Normalyzer’ using PCV and RLE plots respectively. PCV reflects the ability of a normalization method to decrease intragroup variation between technical and/or biological replicates [8]. The results indicated that, VSN-G-normalized data consistently showed lesser intra-group variation in all datasets compared to data normalized by other methods (Fig. 3I). Additionally, in datasets B–F VSN-R normalized data also reduced intra group variation. Further, qualitative assessment of the normalization methods with lowest PCV (VSN-G and VSN-R) by RLE plot indicated that only VSN-G showed good inter and intra group alignment among the replicates in all datasets (Fig. 3II). Thus, VSN-G was identified as the optimal normalization method using ‘Normalyzer’ based evaluation.

### Assessment of VSN-G normalized data by cluster analysis

**Fig. 4:**
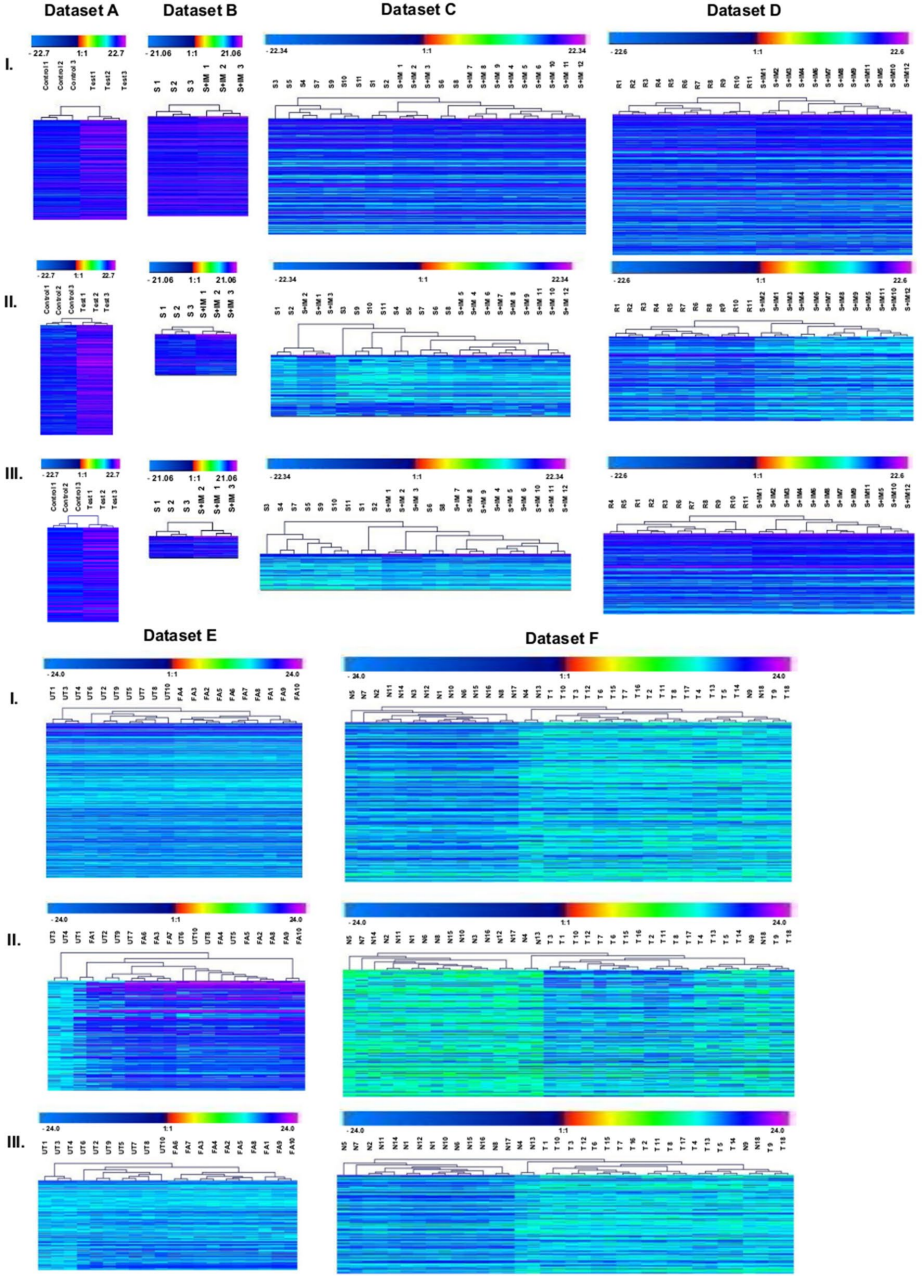
Hierarchical clustering of differentiators obtained from VSN-G normalized data based on **I**- p-value, **II**- fold change, **III**- p- value together with fold change

Differentiators identified from data normalized by VSN-G method based on p-value, fold change and a combination of both were subjected to cluster analysis. Differentiators identified by all three criteria could segregate the comparison groups appropriately in datasets A, B and D but not in dataset C, E and F (Fig. 4). Though VSN-G was identified as optimal normalization method based on PCV and RLE plots, the differentiators identified did not show consistent efficiency in clustering. In order to understand the contribution of VSN-G normalization to improper clustering of datasets C, E and F, differentiators identified by all three criteria, from data normalized with the remaining eleven methods were assessed for their clustering ability. The aim was to detect if any other normalization method could improve segregation of datasets C, E and F while retaining the efficient segregation of datasets A, B and D in VSN-G normalized data.

### Assessment of data normalized by methods other than VSN-G by cluster analysis

**Fig. 5:**
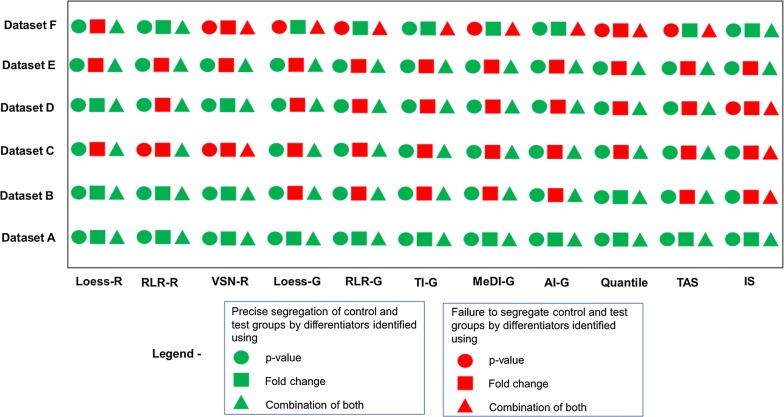
Ability of differentiators to cluster the study groups distinctly

As observed in VSN-G normalized data, clusters obtained from data normalized with the remaining eleven methods yielded improper clustering in datasets E and F. Thus the improper features of clusters i.e. formation of separate cluster by a few normal samples in datasets E and F; segregation of a pair of normal samples (N9 and N18) with tumor samples in dataset F was taken as a consistent feature across normalized data for these two datasets and was not applied to eliminate a cluster as imprecise. While retaining these features, clear segregation of the remaining control and test samples was considered as acceptable clustering efficiency of datasets E and F. Based on this relaxed criteria, it is seen in Fig. 5 (Detailed dendrograms for cluster analysis is given in Additional file 1) and Table 2 that differentiators identified based on p-value efficiently segregate the comparison groups for data normalized by majority of methods. On the other hand, differentiators identified based on fold change could not segregate the comparison groups in majority of the datasets. The ability of differentiators obtained from the combination of p-value and fold-change to segregate sets therefore could be attributed to the influence of p-value. Based on the above experimental evidence p-value is chosen as the criteria for differentiator identification in this study.

**Table 2:** Clustering efficiency of differentiators identified based on p-value, fold change and combination of both, from data normalized by 12 methods

Datasets	Clustering efficiency		
	p-value	Fold change	Both
A	100% (12/12 methods)	100% (12/12 methods)	100% (12/12 methods)
B	100% (12/12 methods)	42% (5/12 methods)	92% (11/12 methods)
C	75% (9/12 methods)	0% (0/12 methods)	75% (9/12 methods)
D	92% (11/12 methods)	25% (3/12 methods)	92% (11/12 methods)
E	100% (12/12 methods)	0% (0/12 methods)	100% (12/12 methods)
F	42% (5/12 methods)	66.6% (8/12 methods)	25% (3/12 methods)

**Fig. 6:**
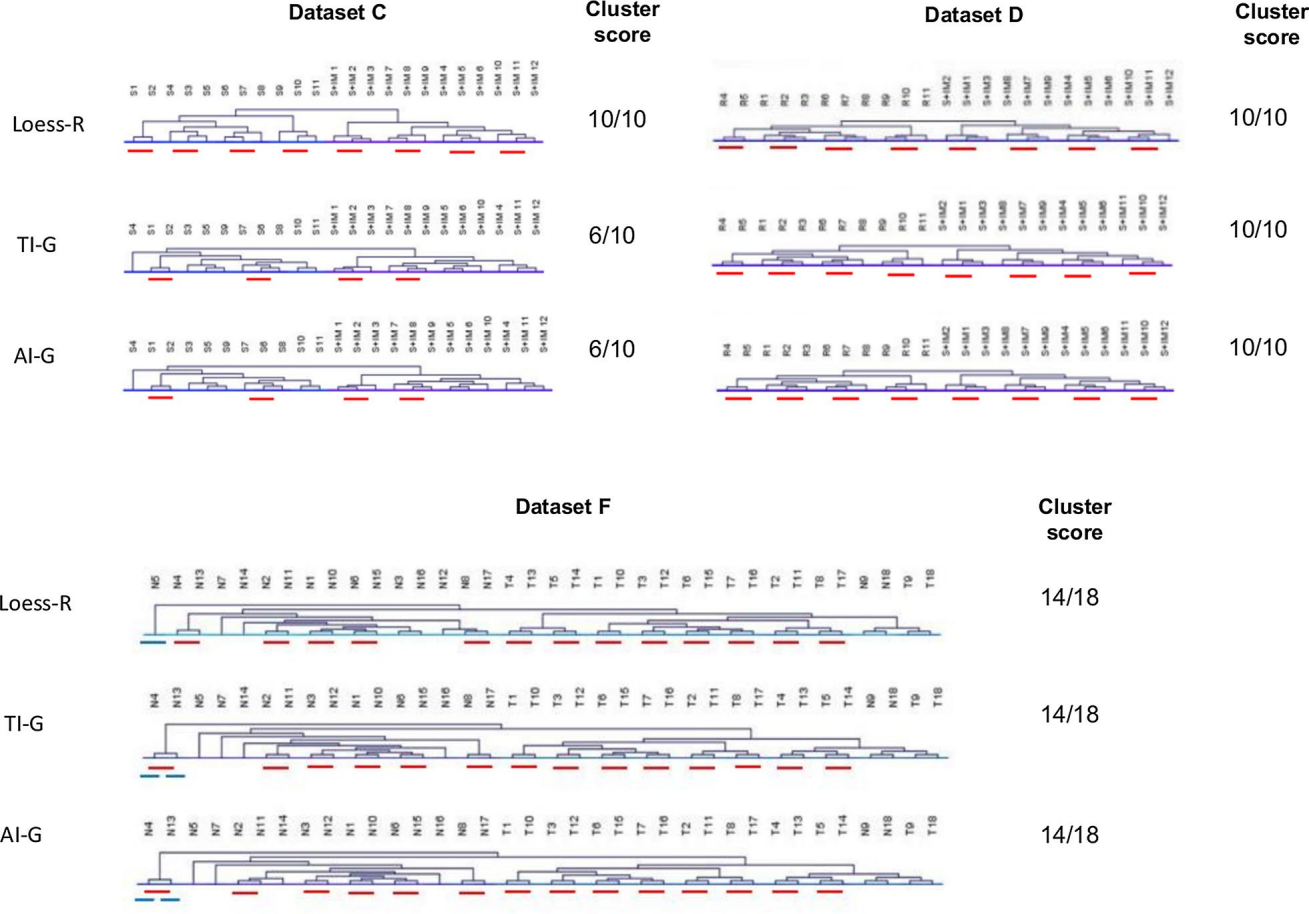
Evaluation of efficiency of p-value based differentiators to sub-stratify the technical replicates. Proper clustering of test and control groups is given a score of 2 and proper sub-clustering of technical replicates of each set indicated by red line, is given a score of 1. In dataset F, a score of -1 is given to each control which formed a cluster outside the major control or test cluster, indicated by blue line

Of the 11 normalization methods assessed, differentiators identified based on p-value from data normalized by 3 methods (Loess-R, TI-G and AI-G) segregated the comparison groups precisely in all datasets (Fig. 5). These were further evaluated using more stringent criteria to identify the most optimal method for biomarker discovery. The criteria was to sub-cluster the technical replicates, of control and test groups, belonging to each biological replicate precisely in datasets C, D and F. Dataset E was not subclustered as each sample was run only once [15]. A scoring system was used to achieve this, wherein the ability to segregate control and test groups was given a score of 2. In dataset F, for every control which segregated separately from the major control cluster, a negative score of 1 was given. Thereafter for every correct subgrouping of the technical replicates of control and test, a score of 1 was given. The total score was calculated as score for precise clustering (2) + score of − 1 for each control which clustered separately from the major control cluster in dataset F (not applicable to other datasets) + score for co-segregation of technical replicates in test and control (1)(Fig. 6).

As mentioned earlier, the efficiency of biomarkers lies in their ability to accurately stratify the heterogenous groups in a given population. It is evident from Fig. 6 that differentiators obtained from Loess-R normalized data could not only stratify the comparison groups precisely, but also had maximum sub-stratification score in the three large datasets assessed, thereby indicating its suitability for biomarker discovery.

## Discussion

This study has addressed two previously unattended issues in analysis of quantitative SWATH-MS data, especially relevant to clinical proteomics—(i) experimental demonstration of ideal method of data normalization which does not diminish the vital features of the data necessary for segregation of comparison groups (ii) experimental verification of criteria for identification of differentiators. Carefully chosen sets of samples, mimicking the biological and experimental variations which can influence the data were included in the study. The reference set from public domain (dataset A) represented a quantitatively defined set wherein the differences in relative proportions of the constituents between samples made fold-differences in protein quantities predictable. The study set (datasets B–D) on the other hand represented the heterogeneity inherent to biological samples as in S cells and those contributed by extraneous manipulations such as treatment with imatinib in S + IM and R cells. The validation set (dataset E and F) were analyzed to confirm the findings obtained in the earlier sets. While analysis of single sets in dataset A and B allowed to evaluate differences between quantitatively defined (dataset A) and undefined set (dataset B), multiple sets in dataset C, D, E and F allowed for evaluation of the consistency of observations within quantitatively undefined sets. Further the reference, study and validation sets differed in the depth of spectral ion library, the choice of calibrants for retention time calibration as well as peak intensities. They are the prototypes of variation of methodologies observed in the reported literature. Analysis of these samples was designed to identify a strategy for normalization of SWATH-MS data which is applicable universally.

The differences in datasets throw light on certain valuable aspects of experimental design. Detection of greater number of proteins in dataset A, E and F as compared to B, C and D could be attributed to deeper spectral ion library. For dataset A, library was generated by pooling individual libraries of constituent human, yeast and *E. coli* peptides while for datasets E and F extensive human protein library was used. It could also be due to use of iRT peptides for retention time calibration in dataset A, E and F which allows high-quality spectral library generation. Retention time calibration of datasets B, C and D had been carried out using spiked peptides of *E. coli* beta galactosidase which span a limited range of retention times. This represents the studies where retention time calibration has been carried out using highly conserved and abundant endogenous peptides or spike-in peptides other than iRT [19, 20].

The extent of variation among un-normalized datasets, when assessed by RLE plot, showed a progressive increase from dataset A to D and F (Fig. 2). This increase in variation could be attributed to increase in sample heterogeneity, as dataset A, generated by addition of defined proportions of constituents, was less heterogenous and had more precise quantification. Dataset B on the other hand, was not a defined set and thus would exhibit variations inherent to any biological system. In datasets C and D, the probability of variation increased as the heterogeneity increased due to inclusion of greater number of samples. Dataset E which also involved cell lines as in datasets C and D, showed least variation which reflects precision in experimentation but is not commonly observed due to experimental errors. Dataset F included human samples which are inherently heterogenous. Such variations are a commonplace in clinical samples and reflect in the assessment of un-normalized data based on RLE plot. These observations emphasized the need for normalization of SWATH-MS data.

In most of the previously reported SWATH-MS studies, data has been normalized by TAS [21–29], median [15, 30–32], TI [33–36], quantile [37–39] and IS [40, 41] methods. In this study, to identify the optimum normalization method, datasets A–F were normalized using 10 normalization methods from Normalyzer and 2 methods from Marker View software, which included the above mentioned methods used in previous studies. Their normalization efficiency in Normalyzer was evaluated based on PCV and RLE plots. VSN-G was found to efficiently normalize not only dataset A with minimum variation but also datasets B-F with considerable variation (Fig. 3). This indicates that VSN-G could have a broad applicability for normalization of SWATH-MS data. VSN normalization has also been reported to efficiently normalize data generated by DIA using LTQ orbitrap [8]. Considering wide use of SWATH-MS for biomarker identification, the utility of VSN-G normalized data for biomarker discovery was assessed based on its ability to yield differentiators which segregate the comparison groups precisely.

Differentiators could be identified by comparing quantities of proteins in comparison groups based on p value, fold change or combination of both. PubMed search revealed that in over 134 human SWATH related publications in a span of 5 years (2012-Mar 2018), 45 studies (33.6%) were aimed at identifying differentiators between control and test groups. Out of these 45 studies, we observed that 14 studies (31.1%) used statistical significance (p-value), 6 studies (13.3%) used fold change and 25 studies (55.6%) used both (p value and fold change) as criteria to identify differentiators. While experimental evidence for either choosing the criteria for differentiator identification (p-value, fold change or combination of both) [42, 43] or their cut-off values [44] is available for transcriptomic data, no such studies are reported for MS data. Our study provides the first experimental evaluation of choice of criteria to identify differentiators from SWATH-MS data for biomarker discovery based on their ability to segregate comparison groups- an essential feature of biomarkers, by cluster analysis. Cluster analysis revealed that differentiators identified based on p-value, from data normalized by 12 methods, could segregate the comparison groups with maximum efficiency in 5 out of 6 datasets (Fig. 5). Hence p-value was chosen as the criteria for differentiator identification in this study.

**Fig. 7:**
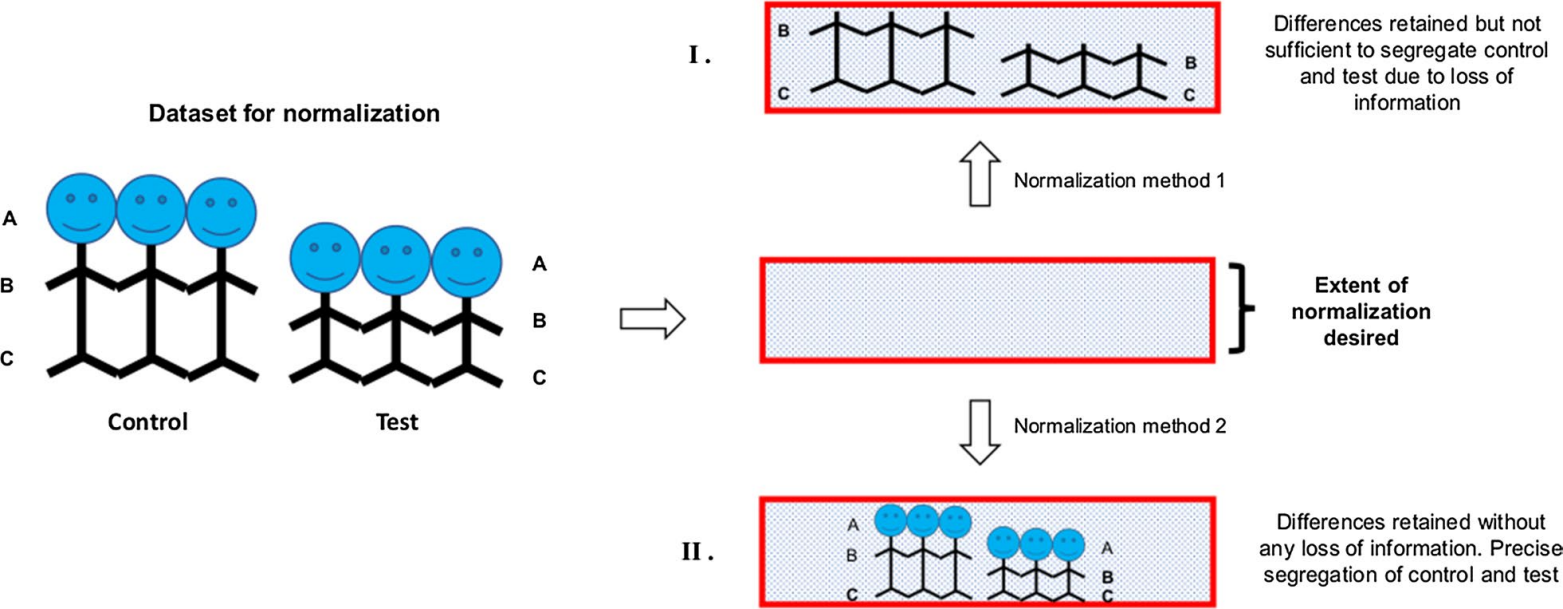
Should application dictate the choice of normalization method for SWATH-MS data?

VSN-G, though was identified as optimal normalization method based on PCV and RLE plots, the differentiators identified did not efficiently cluster the comparison groups in all datasets (Fig. 4), thereby raising question on its suitability for biomarker discovery. Loess-R, a method ranked lower in ‘Normalyzer’ based evaluation, on the other hand, yielded differentiators with maximum clustering as well as sub-clustering efficiency in all datasets assessed (Fig. 6), thereby making it suitable for biomarker discovery by SWATH-MS. This may be due to the differences in the assumptions made for normalization by these methods. The perceived treatment of data is depicted in Fig. 7. VSN-G aims at making the sample variances non-dependent on their mean intensity thereby bringing the samples onto the same scale. This assumption remarkably reduces the intensity differences between samples so as to achieve optimum normalization. However, the reduction in intensity differences is not conducive to identification of differentiators and in turn segregation of comparison groups (Fig. 7I) Loess normalization on the other hand, probably retains the differences between intragroup protein intensities by assuming non-linear relationship between biases in the data and the magnitude of protein intensity—a feature essential for segregation of comparison groups (Fig. 7II).

We thus propose that apart from statistically recommended criteria for evaluation of methods for normalization, a biologically relevant criteria like precise stratification of data should be assessed before a normalization method is used for biomarker identification from SWATH-MS data.

## Conclusion

This study for the first time has identified VSN-G as method for optimum normalization of SWATH-MS data based on statistical criteria. Acknowledging the extensive use of this technology for biomarker discovery this study has also identified the normalization strategies conducive to this application. In the process, p-value based identification of differentiators has been demonstrated to be most suitable for biomarker discovery from SWATH-MS data. While VSN-G normalization was not found conducive to biomarker discovery in this study, Loess-R normalization was observed to retain features of the data necessary to yield differentiators which could segregate the comparison groups efficiently. The probable effect of two normalization methods on the data which are responsible for these observations are depicted in Fig. 7. The study has thus demonstrated the need to base the choice of normalization method on the application of the data.

## Additional file

**Additional file 1.** Dendrograms representing hierarchical clustering of control and test groups based on differentiators obtained by p-value, fold change or both in both datasets.

## Data Availability

The proteomics data generated for the study set in the current study have been deposited to the Proteome Xchange Consortium via the PRIDE [45] partner repository with the dataset identifier PXD009686. The reference dataset deposited by Navarro et al. [12], used for analysis in this study, has been obtained from Proteome Xchange Consortium (Identifier—PXD002952).

## Abbreviations

LC–MS: liquid chromatography Mass spectrometry
SWATH: Sequential window acquisition of all theoretical fragment ion spectra
DIA: data independent acquisition
IDA: information/data dependent acquisition
IM: imatinib
K562 S: imatinib sensitive cells
K562 S + IM: sensitive cells treated with imatinib
K562 R: cells resistant to imatinib
DTT: dithiothreitol
IAA: iodoacetamide
FA: formic acid
β-gal: *E. coli* beta galatosidase
ACN: acetonitrile
iRT: indexed retention time
FDR: false discovery rate
XIC: extracted ion chromatogram
R: local normalization
G: global normalization
RLR: robust linear regression
VSN: variance stabilizing normalization
TI: total intensity
AI: average intensity
MedI-G: median intensity
TAS: total area sums
IS: internal standard
PCV: pooled intragroup coefficient of variation
RLE: relative log expression
