# Supplementary material for: Clinical biomarker discovery by SWATH-MS based label-free quantitative proteomics: impact of criteria for identification of differentiators and data normalization method
Source: J Transl Med. 2019 May 31;17:184. doi: 10.1186/s12967-019-1937-9 (PMC6545036; doi:10.1186/s12967-019-1937-9)
Supplement: Supplementary file 1 — Additional file 1. Dendrograms representing hierarchical clustering of control and test groups based on differentiators obtained by p-value, fold change or both in both datasets. [file 12967_2019_1937_MOESM1_ESM.docx]

**ADDITIONAL FILE 1**

**Clinical biomarker discovery by SWATH-MS-based label-free quantitative proteomics: impact of criteria for identification of differentiators and data normalization method**

*Mythreyi Narasimhan^1,2^, Sadhana Kannan^1^, Aakash Chawade^3^, Atanu Bhattacharjee^4^, Rukmini Govekar^1,2^**

^1^Advanced Centre for Treatment, Research and Education in Cancer, Tata Memorial Centre, Kharghar, Navi Mumbai 410610, India.

^2^Homi Bhabha National Institute, BARC Training School Complex, Anushakti Nagar, Mumbai 400094, India.

^3^Department of Plant Breeding, Swedish University of Agricultural Sciences, Alnarp, Sweden.

^4^Section of Biostatistics, Centre for Cancer Epidemiology, Tata Memorial Centre, Kharghar, Navi Mumbai 410610, India.

*Email address:* [*mnarasimhan@actrec.gov.in*](mailto:mnarasimhan@actrec.gov.in)*;* [*skannan@actrec.gov.in*](mailto:skannan@actrec.gov.in)*;* [*aakash.chawade@slu.se*](mailto:aakash.chawade@slu.se)*;* [*abhattacharjee@actrec.gov.in*](mailto:abhattacharjee@actrec.gov.in)*;* [*rgovekar@actrec.gov.in*](mailto:rgovekar@actrec.gov.in)

*corresponding author


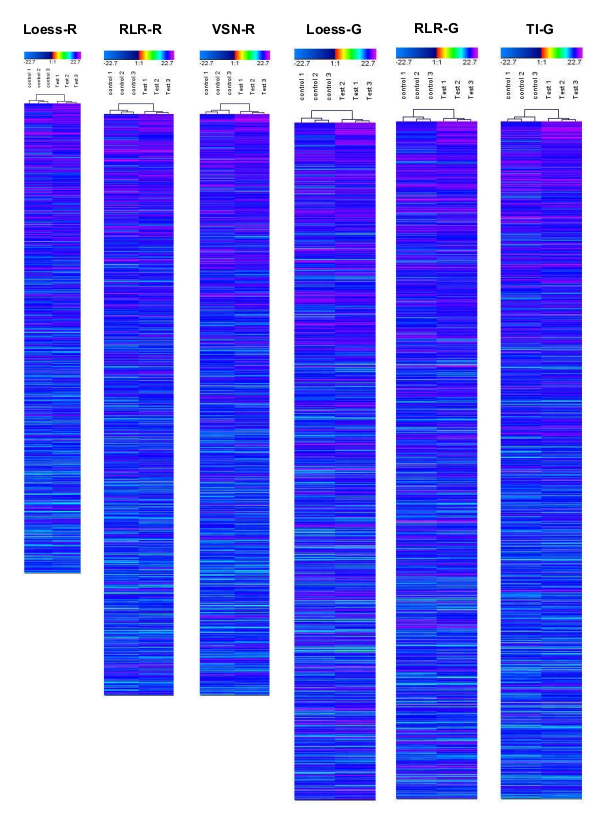


**Figure S1 – Dendrograms representing hierarchical clustering of control and test groups of dataset A based on differentiators identified using p-value.**


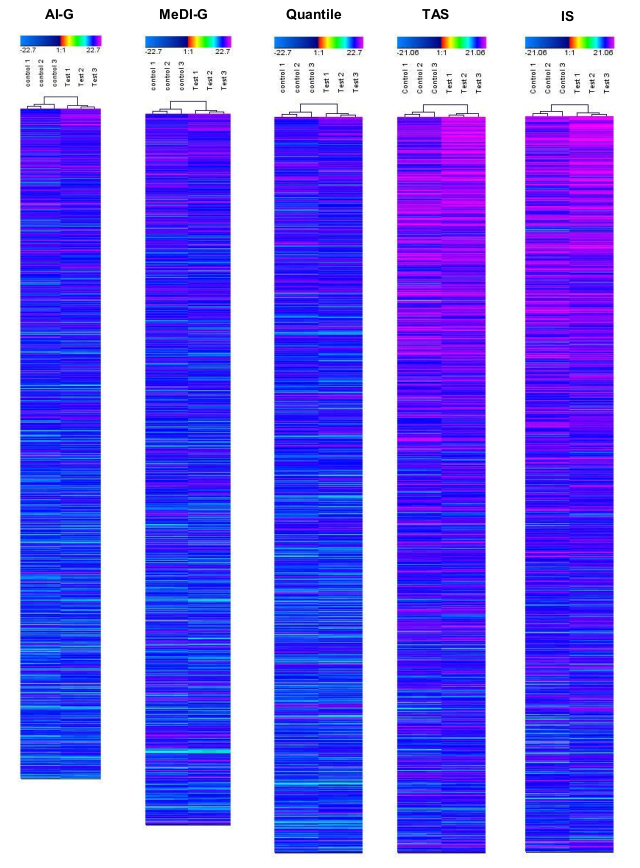


**Figure S1 (contd.) - Dendrograms representing hierarchical clustering of control and test groups of dataset A based on differentiators identified using p-value.**


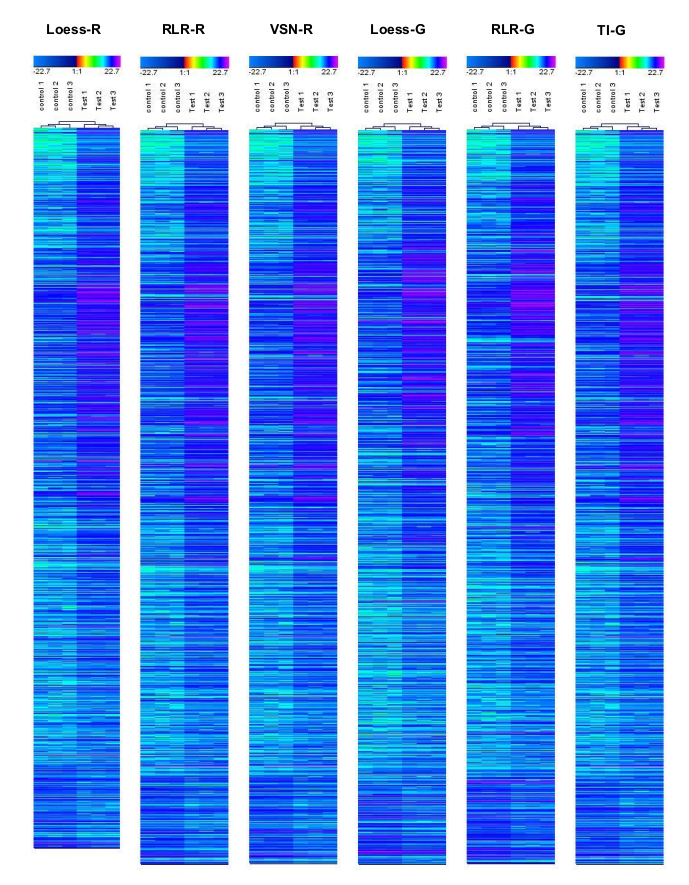


**Figure S2 – Dendrograms representing hierarchical clustering of control and test groups of dataset A based on differentiators identified using fold change.**


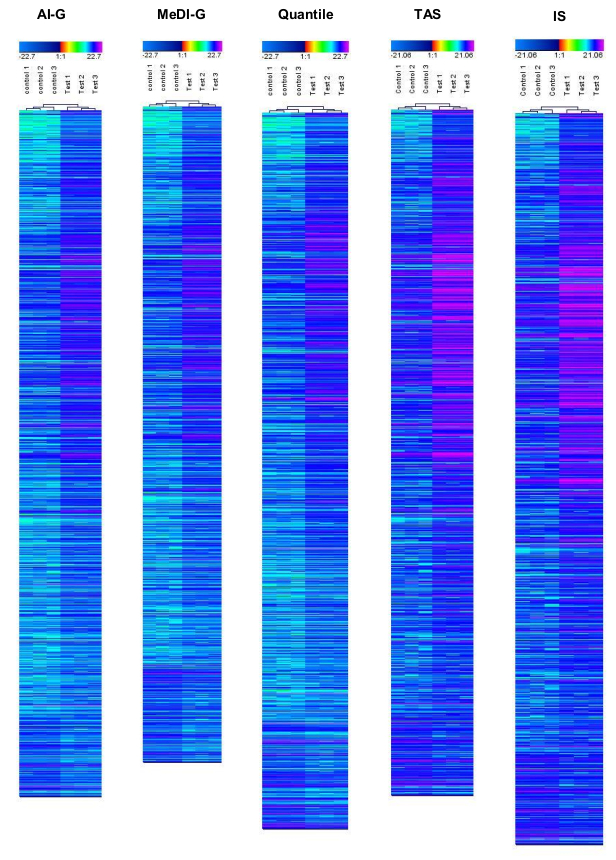


**Figure S2 (contd.) – Dendrograms representing hierarchical clustering of control and test groups of dataset A based on differentiators identified using fold change.**


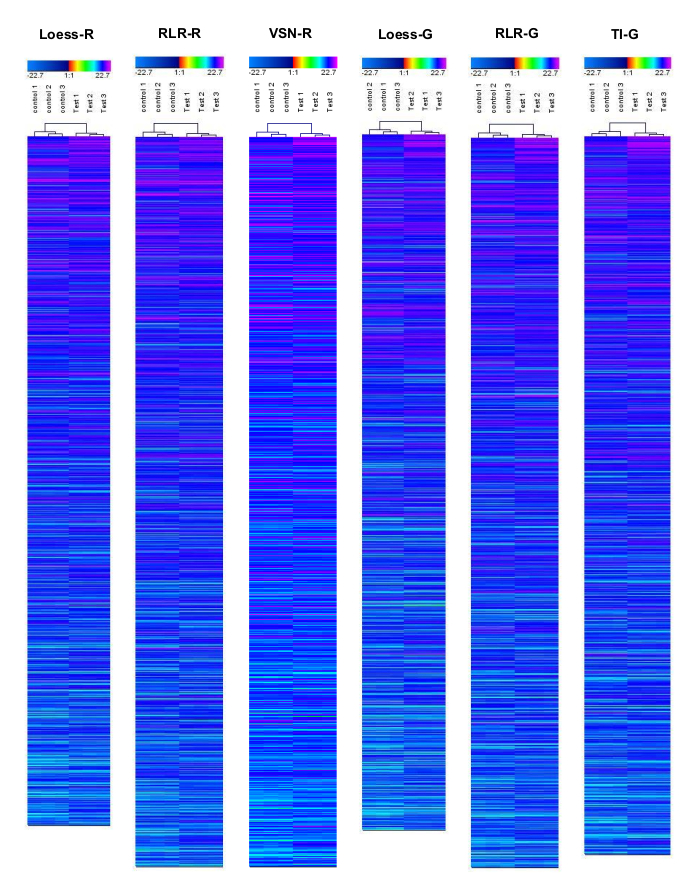


**Figure S3 – Dendrograms representing hierarchical clustering of control and test groups of dataset A based on differentiators identified using both p-value & fold change.**


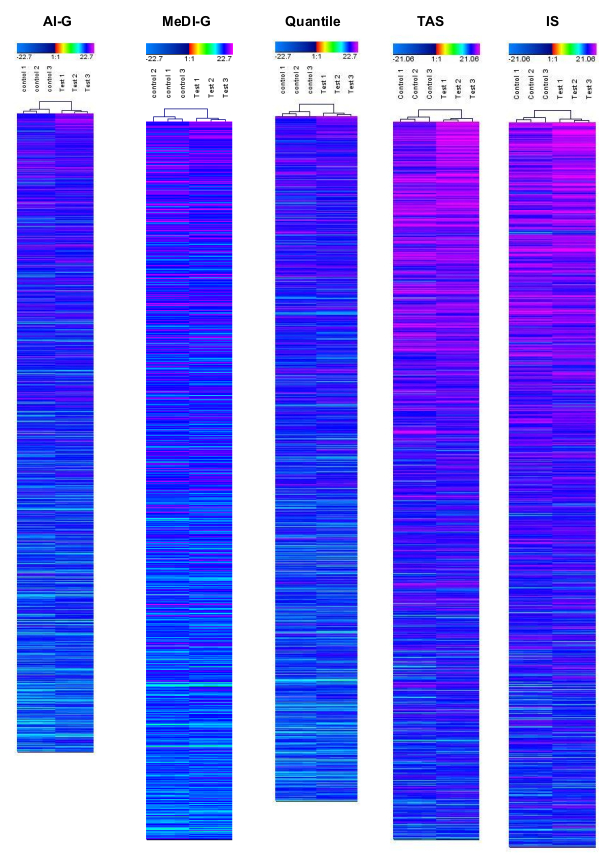


**Figure S3 (contd.) - Dendrograms representing hierarchical clustering of control and test groups of dataset A based on differentiators identified using both p-value & fold change.**

**
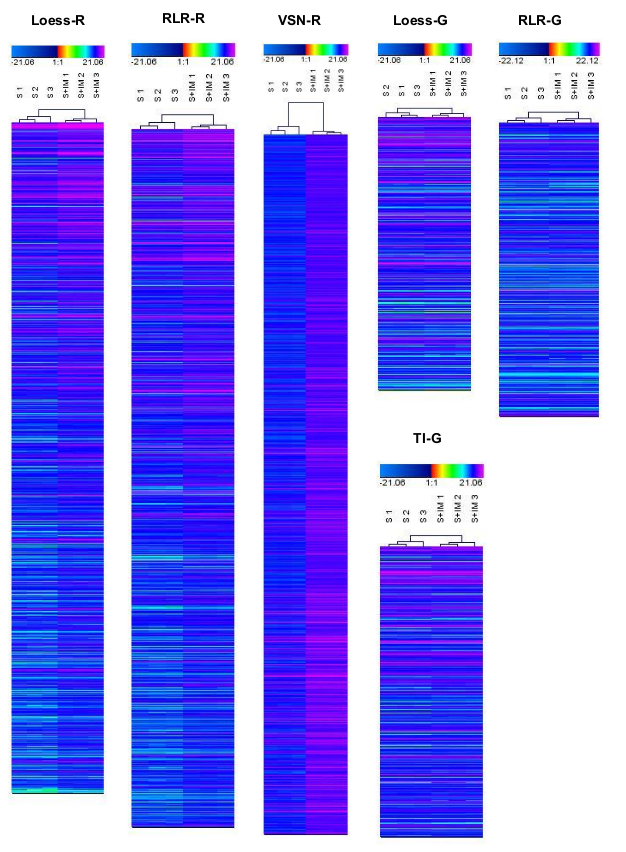
**

**Figure S4 – Dendrograms representing hierarchical clustering of control (S) and test (S+IM) groups of dataset B based on differentiators identified using p-value.**


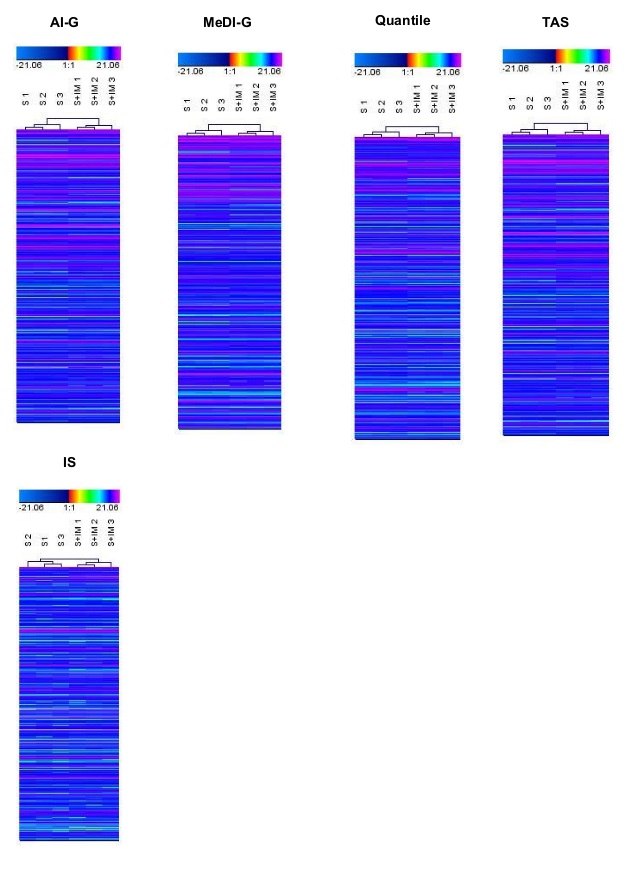


**Figure S4 (Contd.) – Dendrograms representing hierarchical clustering of control (S) and test (S+IM) groups of dataset B based on differentiators identified using p-value.**


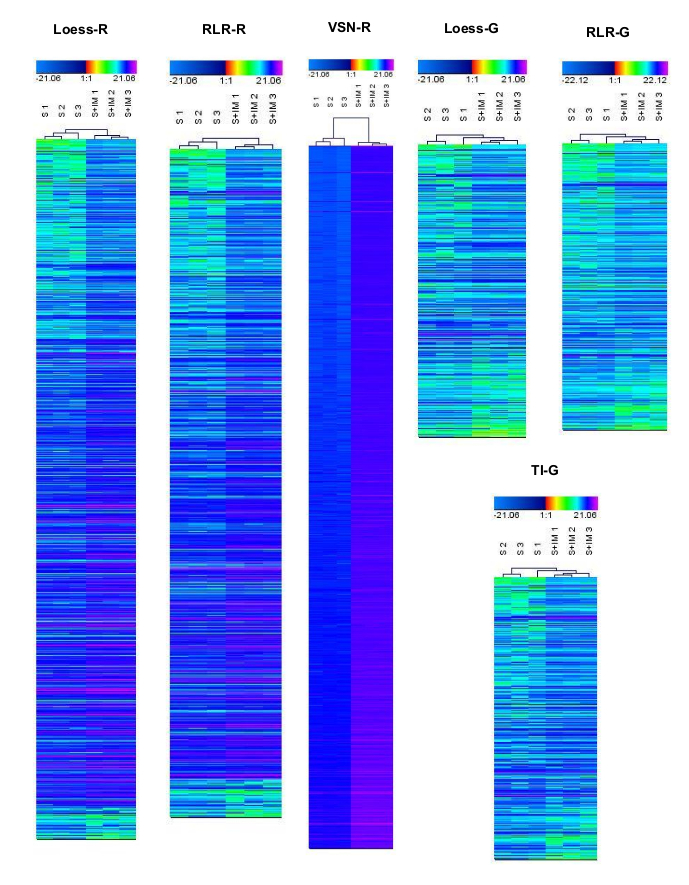


**Figure S5 – Dendrograms representing hierarchical clustering of control (S) and test (S+IM) groups of dataset B based on differentiators identified using fold change.**


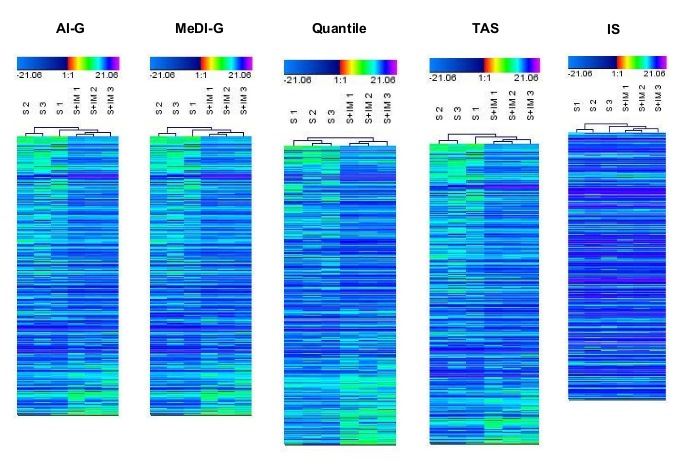


**Figure S5 (contd..) – Dendrograms representing hierarchical clustering of control (S) and test (S+IM) groups of dataset B based on differentiators identified using fold change.**


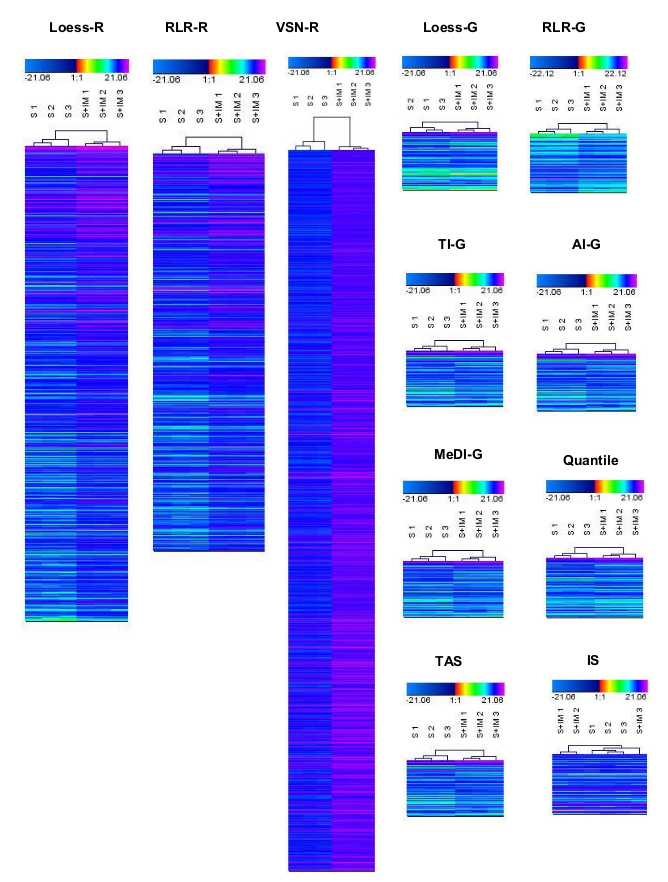


**Figure S6 – Dendrograms representing hierarchical clustering of control (S) and test (S+IM) groups of dataset B based on differentiators identified using both p-value and fold change.**


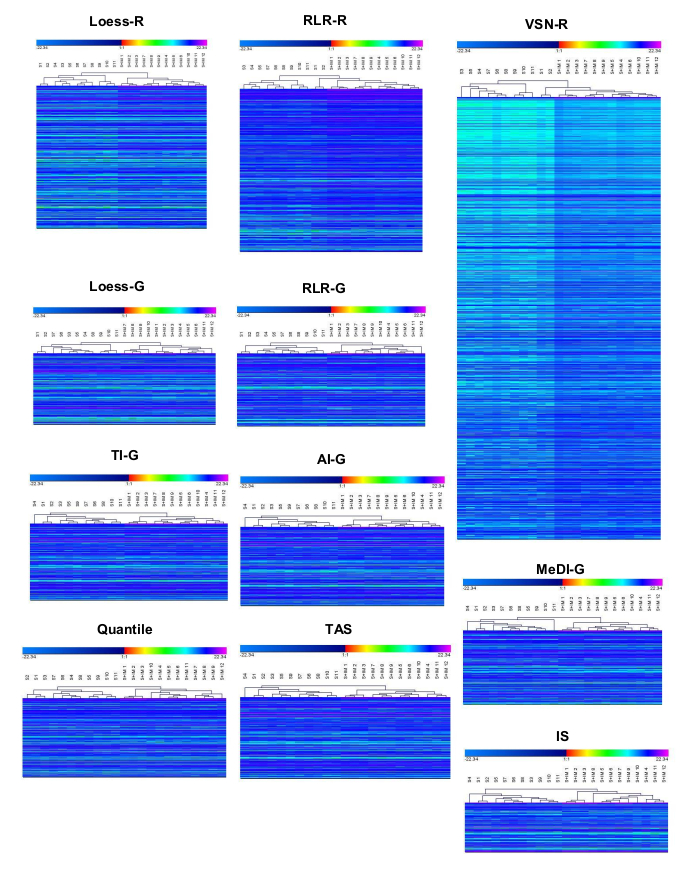


**Figure S7 – Dendrograms representing hierarchical clustering of control (S) and test (S+IM) groups of dataset C based on differentiators identified using p-value.**


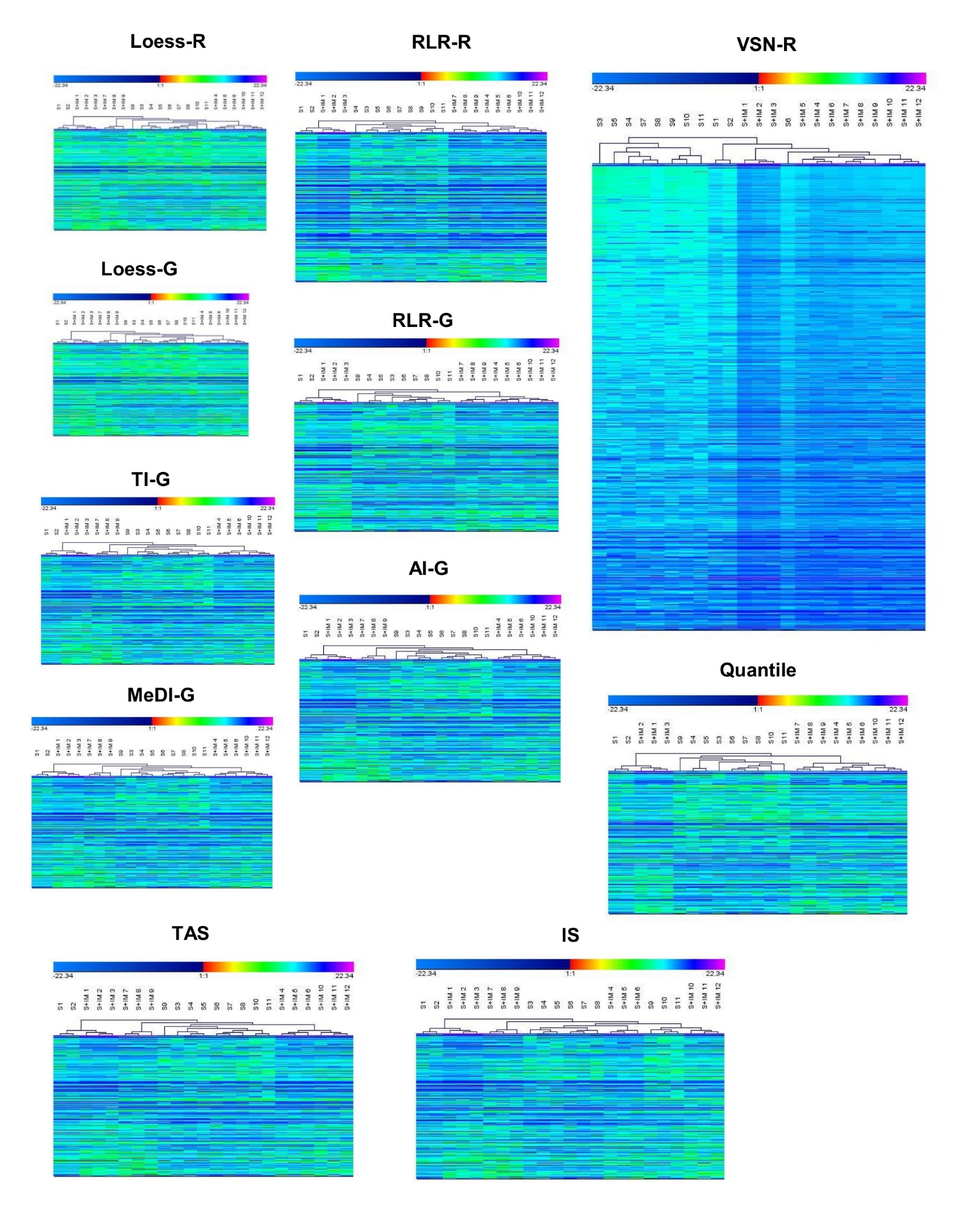


**Figure S8 – Dendrograms representing hierarchical clustering of control (S) and test (S+IM) groups of dataset C based on differentiators identified using fold change.**


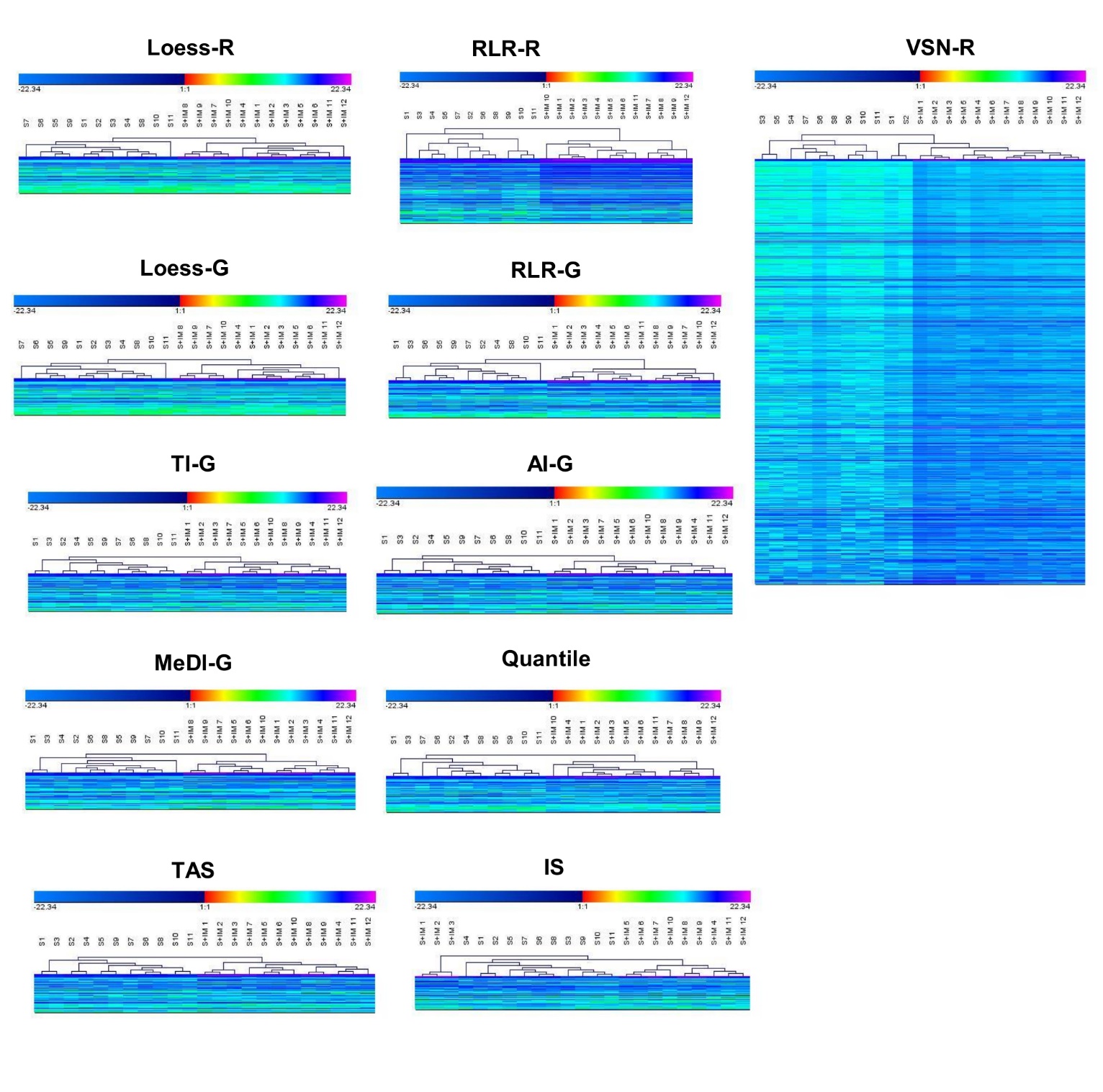


**Figure S9 – Dendrograms representing hierarchical clustering of control (S) and test (S+IM) groups of dataset C based on differentiators identified using both p-value and fold change.**


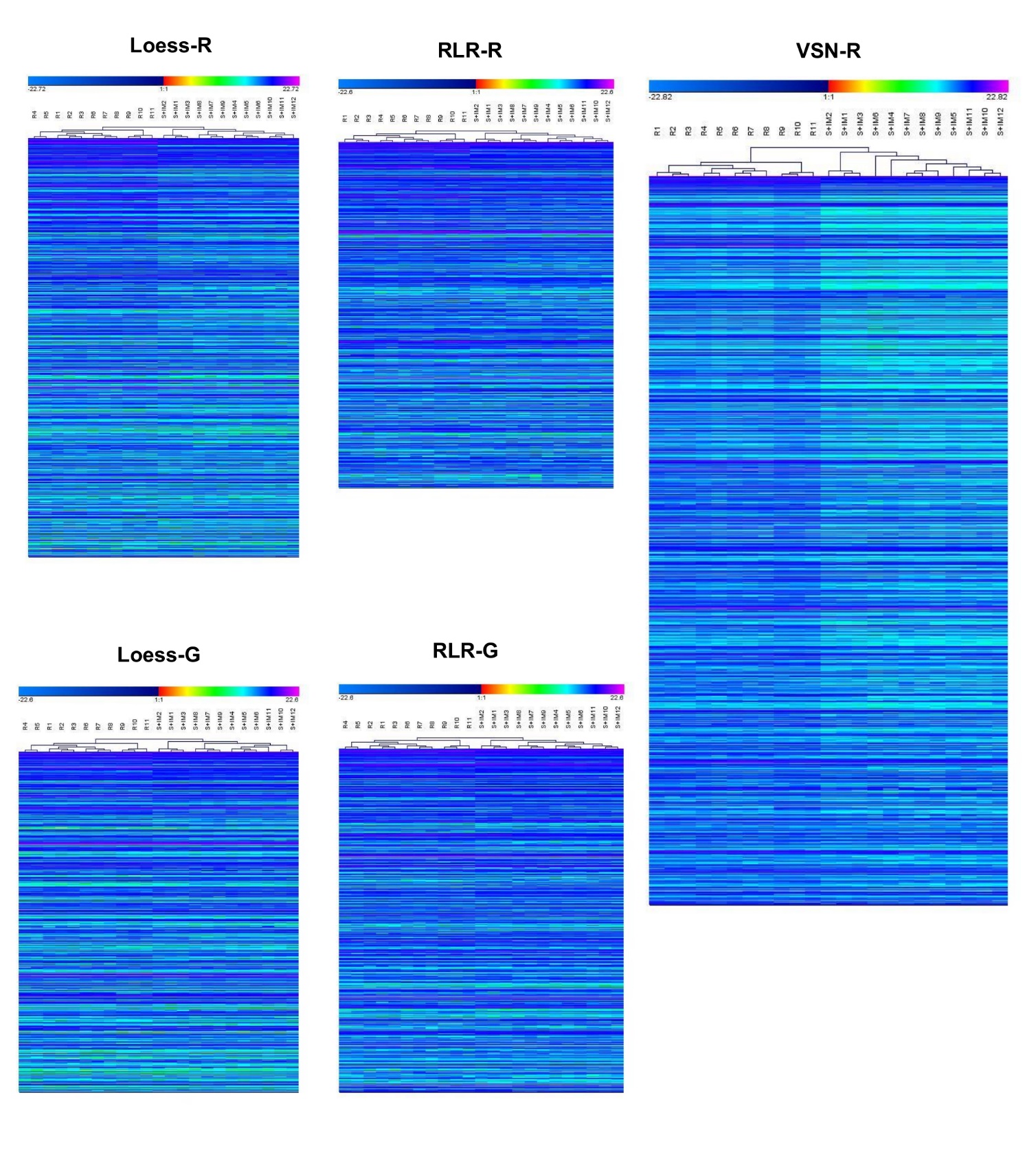


**Figure S10 – Dendrograms representing hierarchical clustering of control (S+IM) and test (R) groups of dataset D based on differentiators identified using p-value.**

**
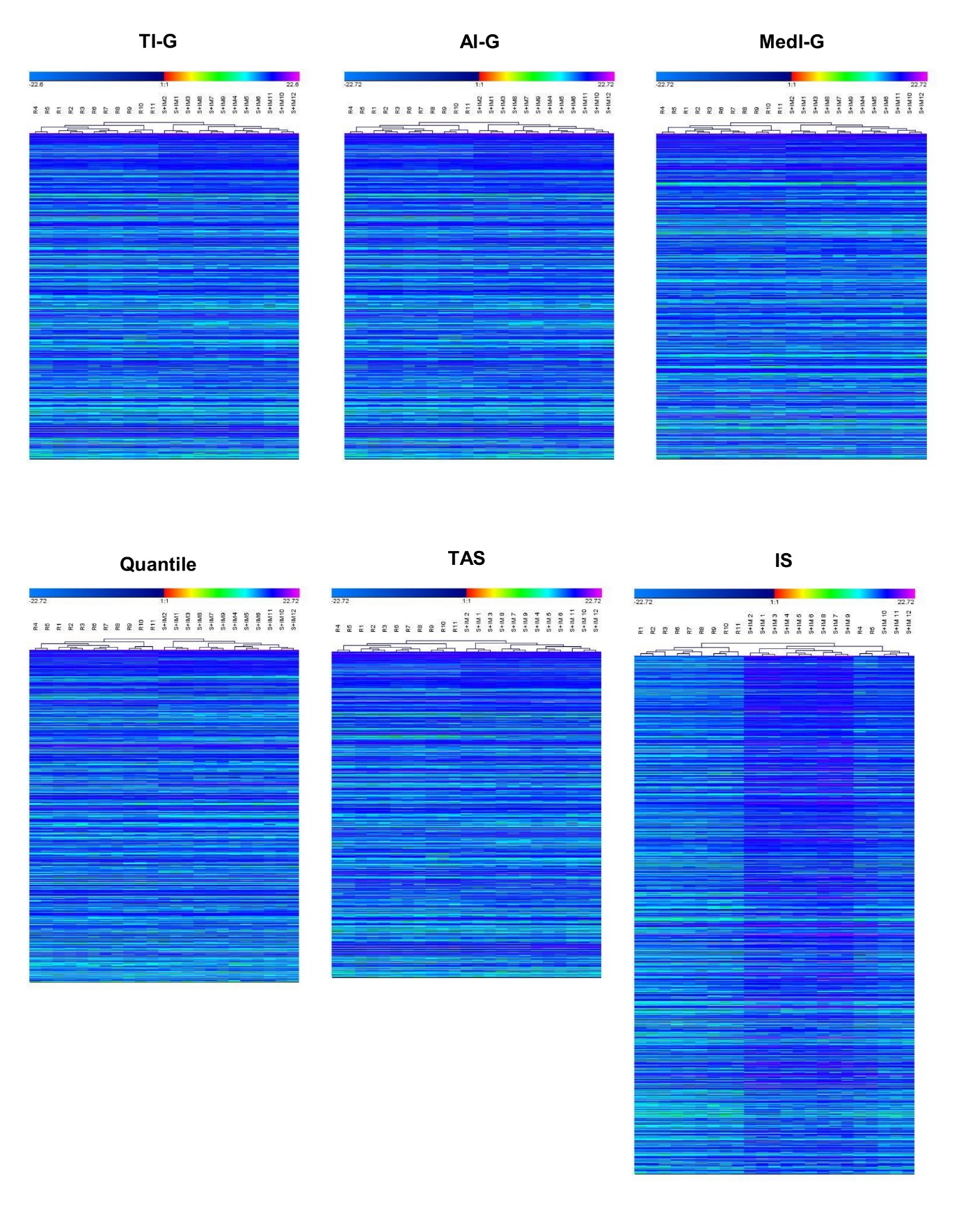
**

**Figure S10 (contd…) – Dendrograms representing hierarchical clustering of control (S+IM) and test (R) groups of dataset D based on differentiators identified using p-value.**

**
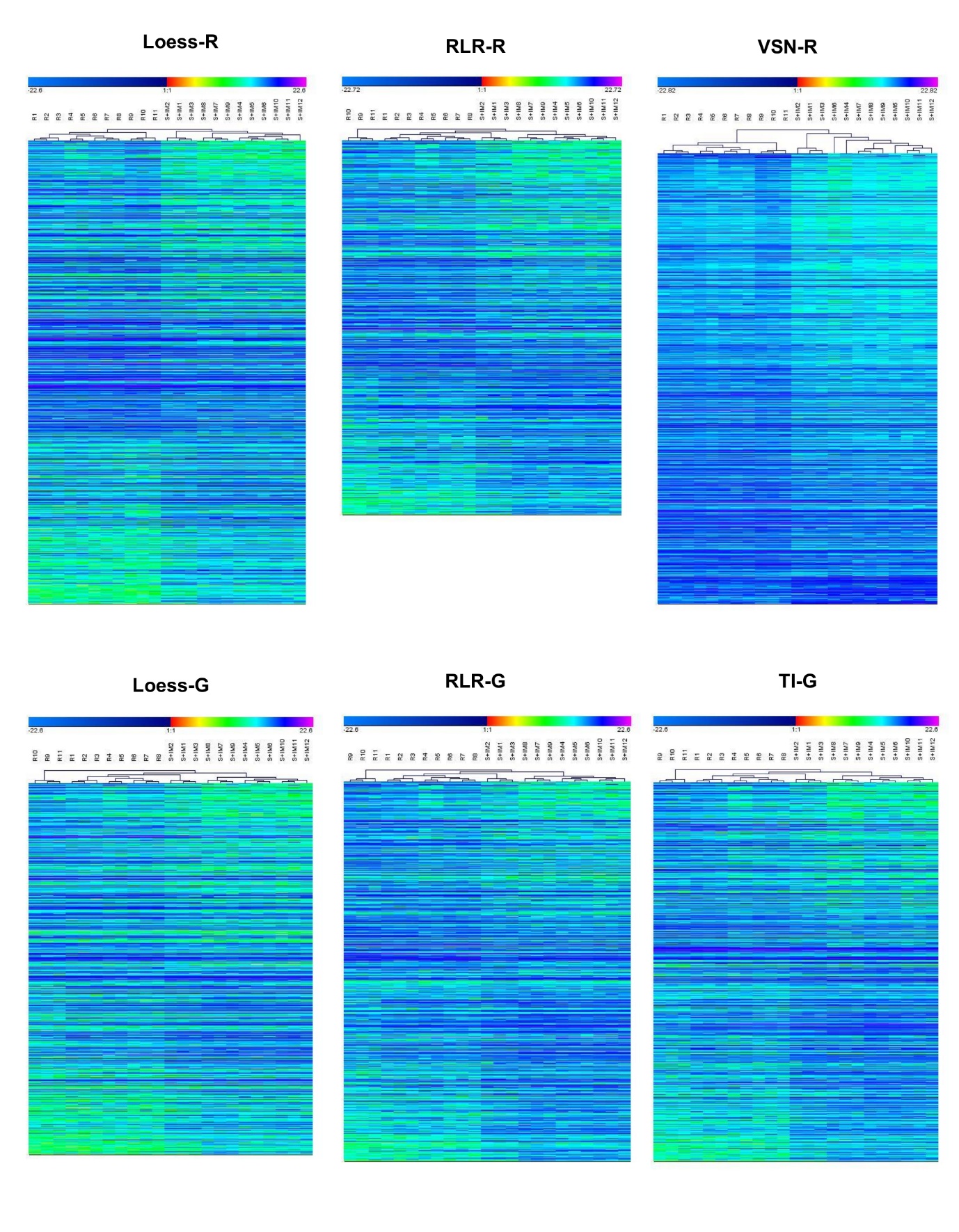
**

**Figure S11 – Dendrograms representing hierarchical clustering of control (S+IM) and test (R) groups of dataset D based on differentiators identified using fold change.**

**
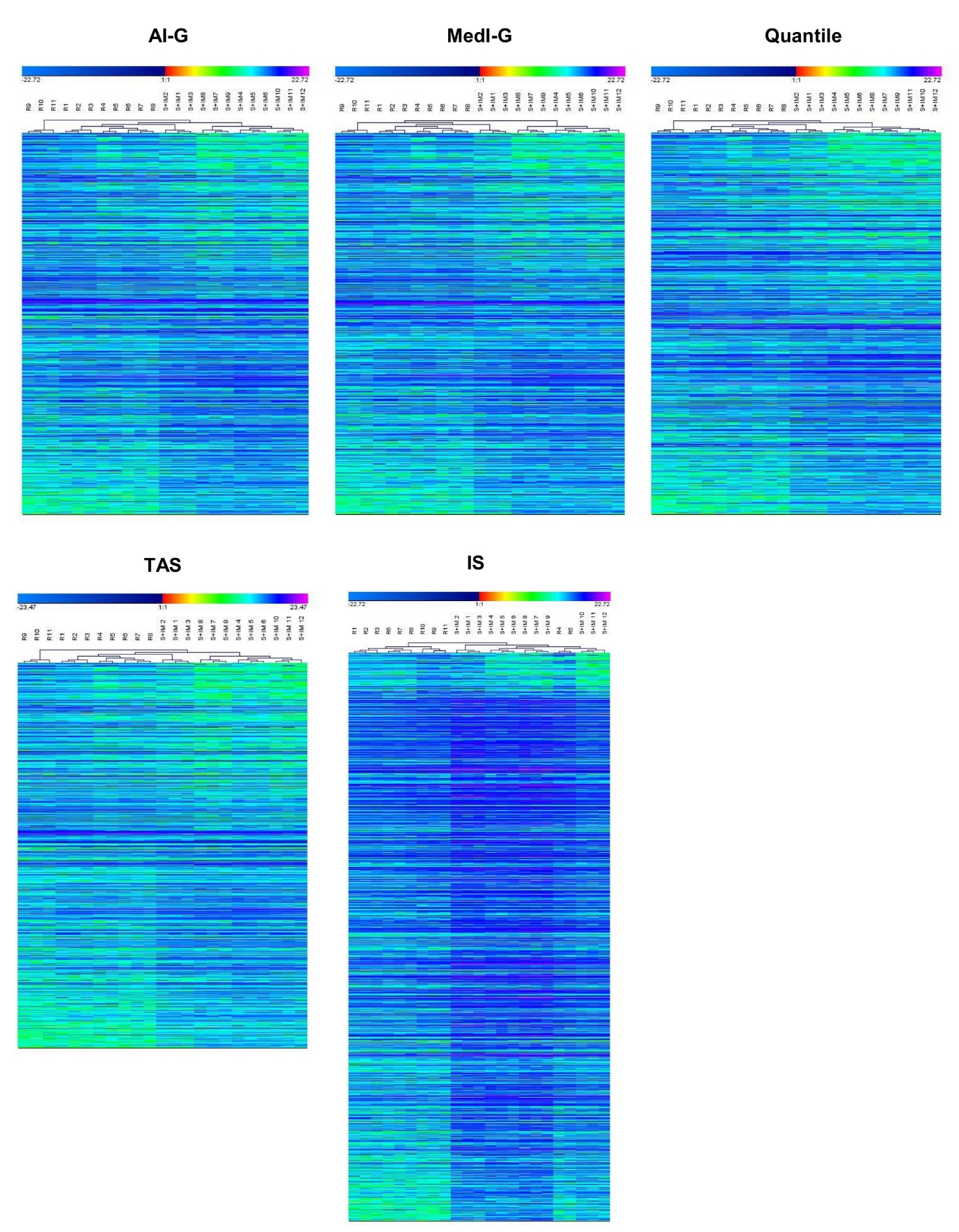
**

**Figure S11 (contd..) – Dendrograms representing hierarchical clustering of control (S+IM) and test (R) groups of dataset D based on differentiators identified using fold change.**

**
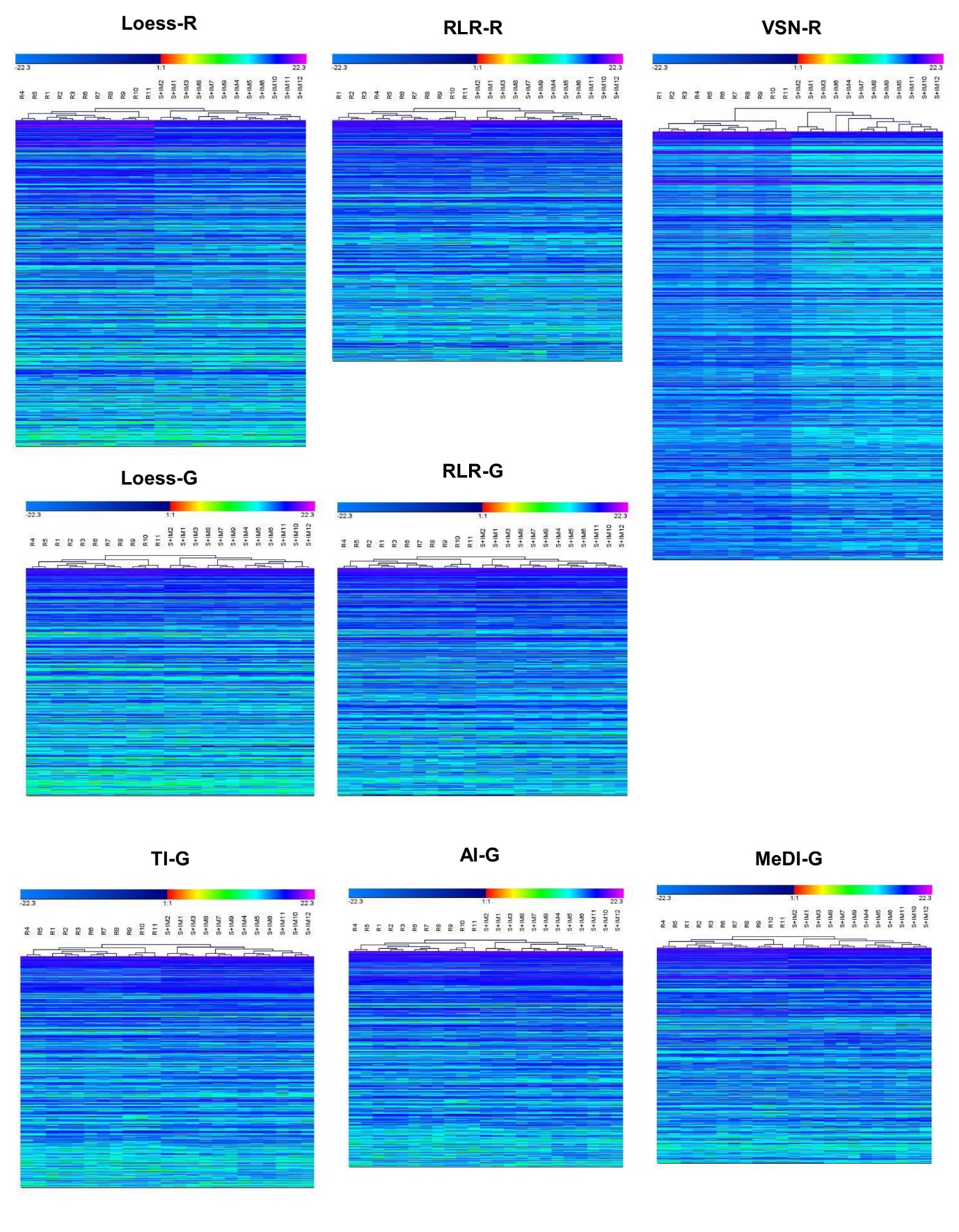
**

**Figure S12 – Dendrograms representing hierarchical clustering of control (S+IM) and test (R) groups of dataset D based on differentiators identified using both p-value & fold change.**

**
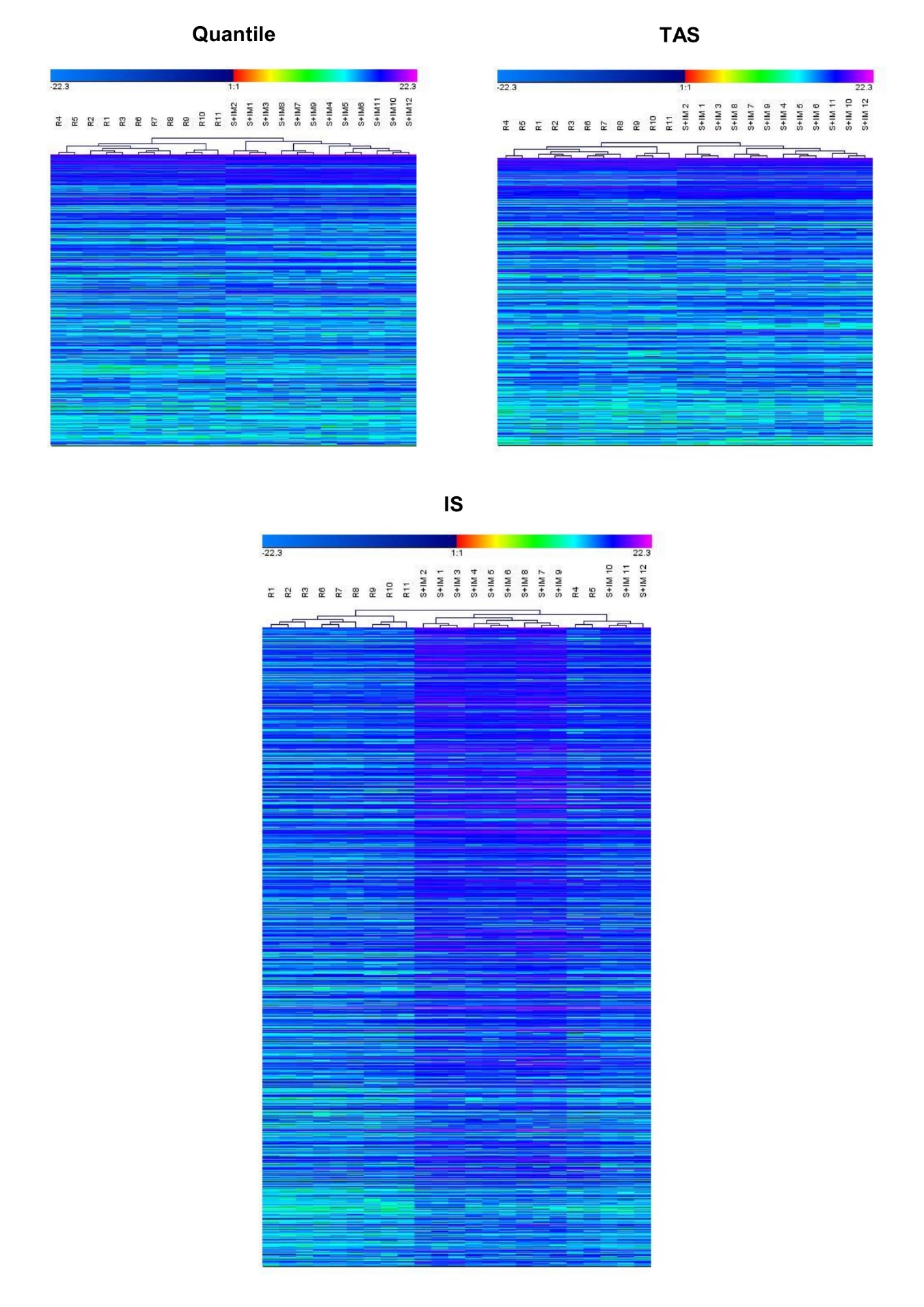
**

**Figure S12 (contd..)– Dendrograms representing hierarchical clustering of control (S+IM) and test (R) groups of dataset D based on differentiators identified using both p-value & fold change.**

**
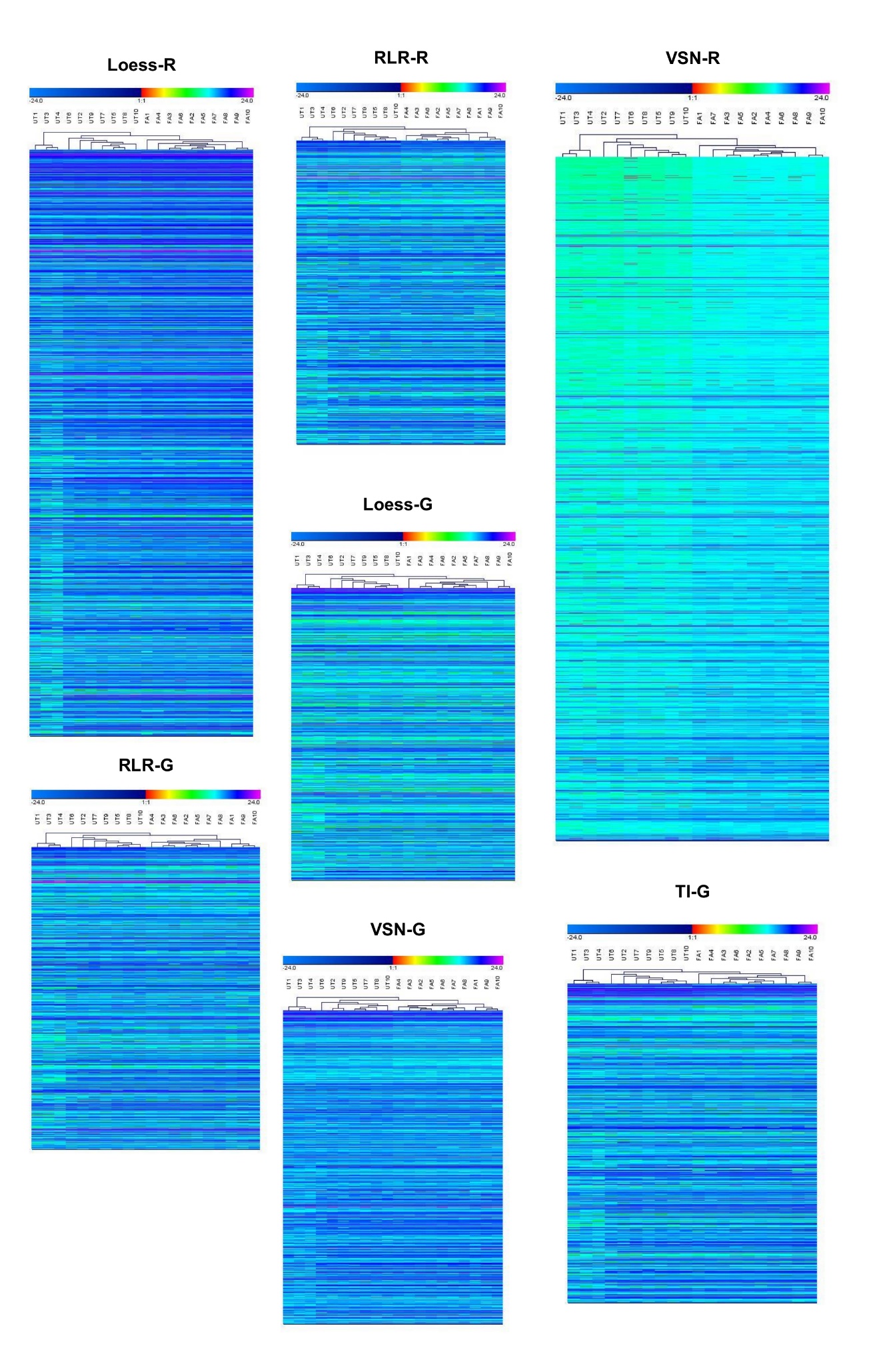
**

**Figure S13 – Dendrograms representing hierarchical clustering of control (UT) and test (FA) groups of dataset E based on differentiators identified using p-value.**


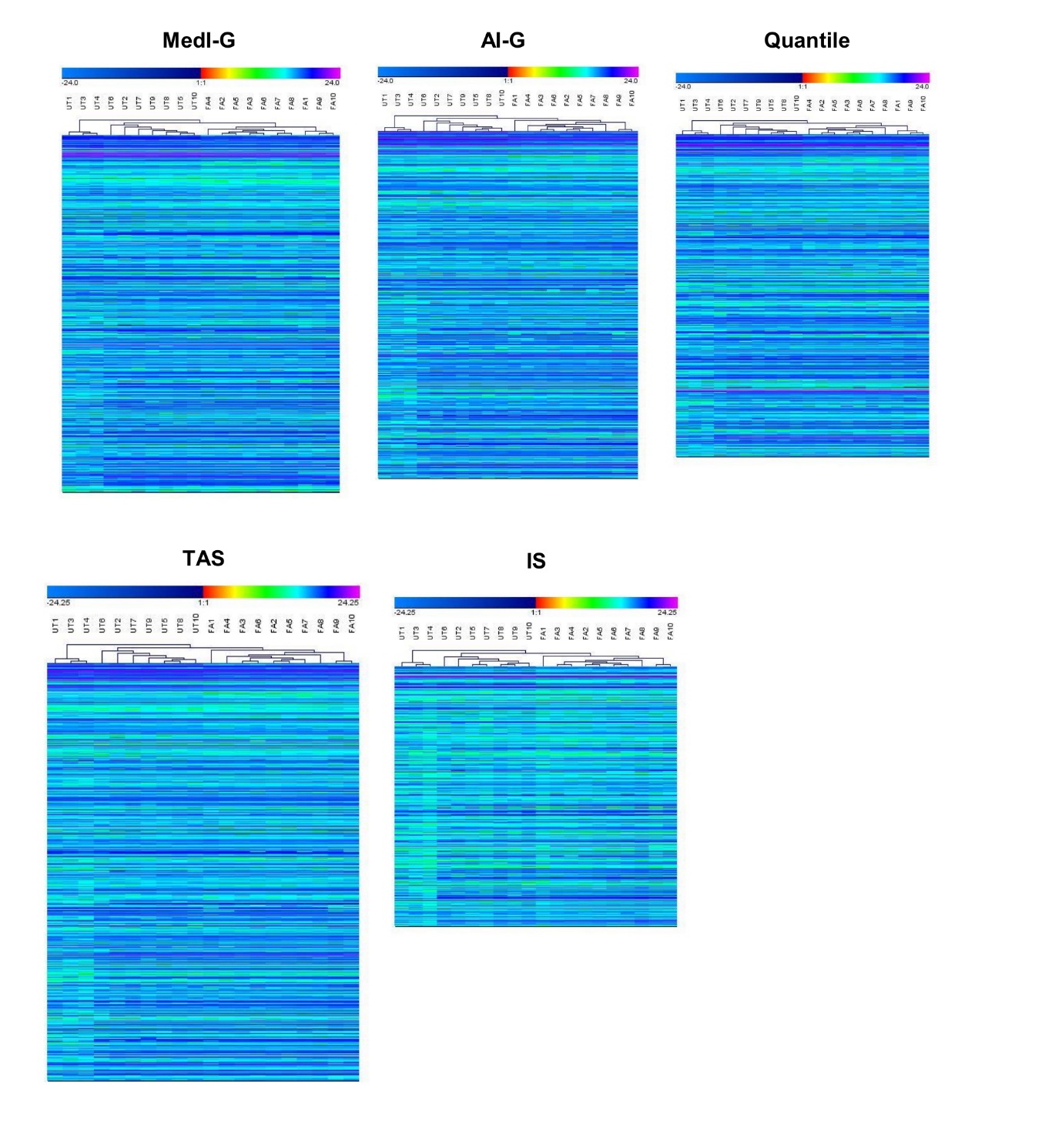


**Figure S13(contd..) – Dendrograms representing hierarchical clustering of control (UT) and test (FA) groups of dataset E based on differentiators identified using p-value.**


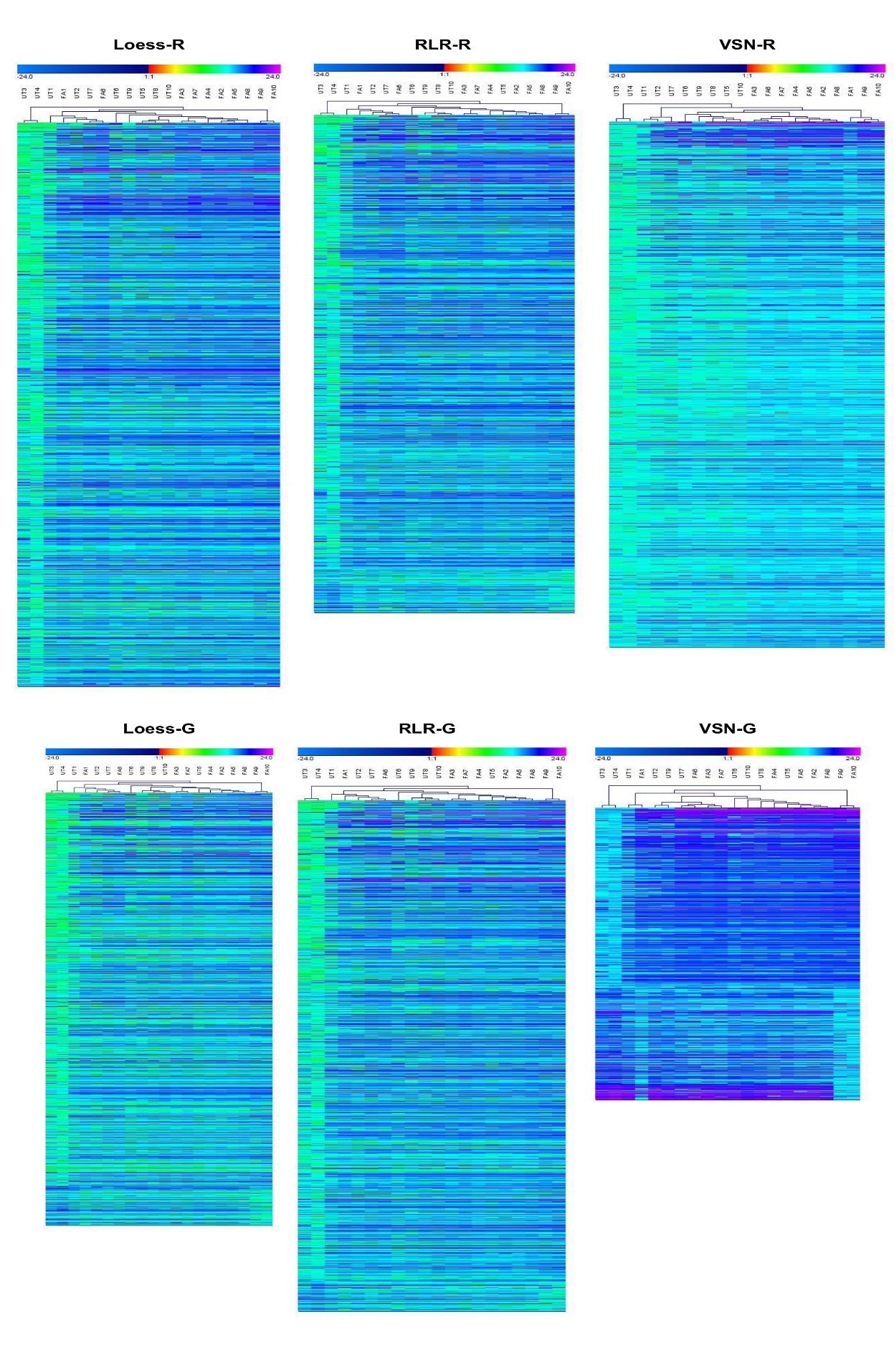


**Figure S14 – Dendrograms representing hierarchical clustering of control (UT) and test (FA) groups of dataset E based on differentiators identified using fold change.**


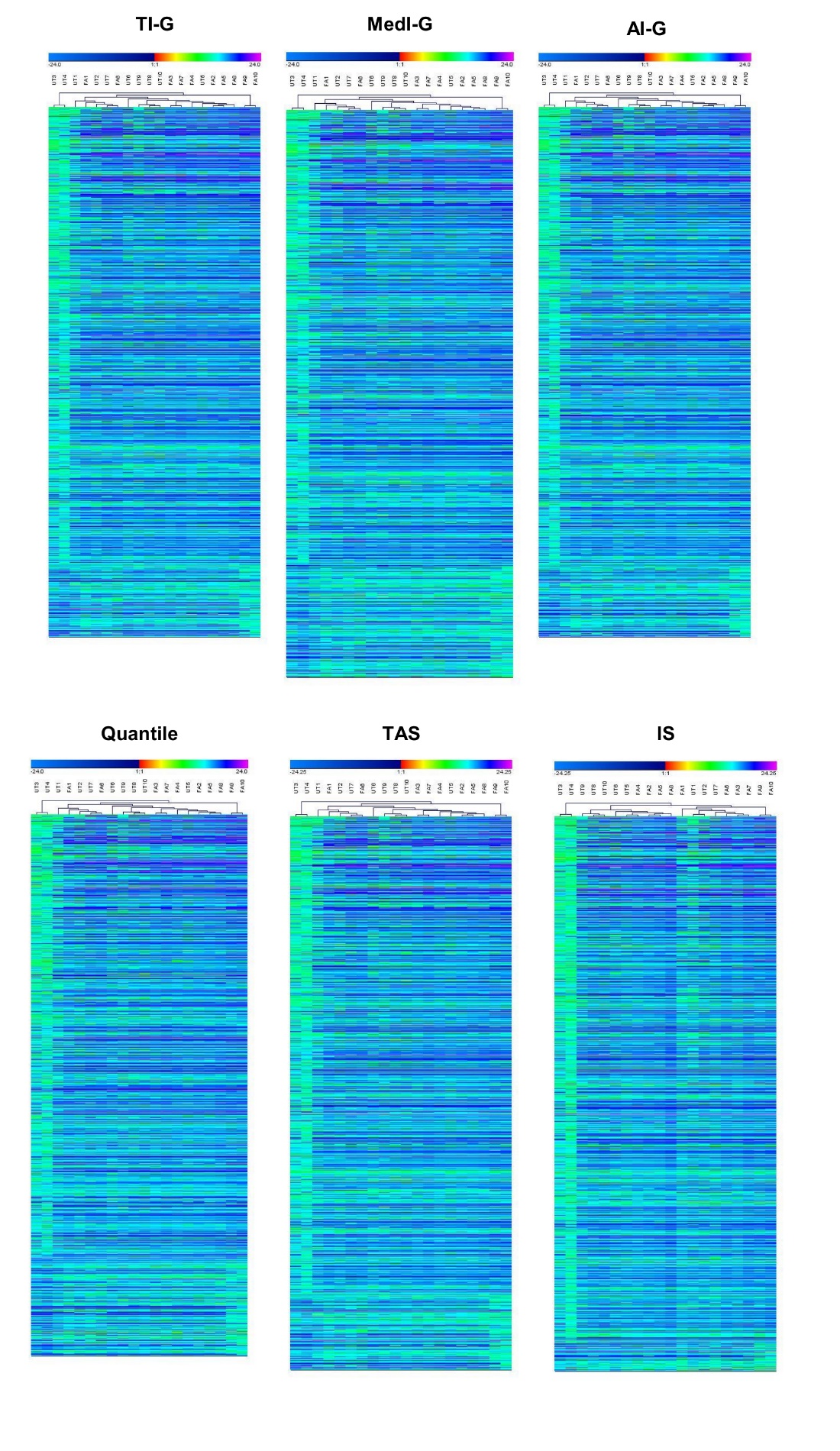


**Figure S14 (contd.) – Dendrograms representing hierarchical clustering of control (UT) and test (FA) groups of dataset E based on differentiators identified using fold change.**


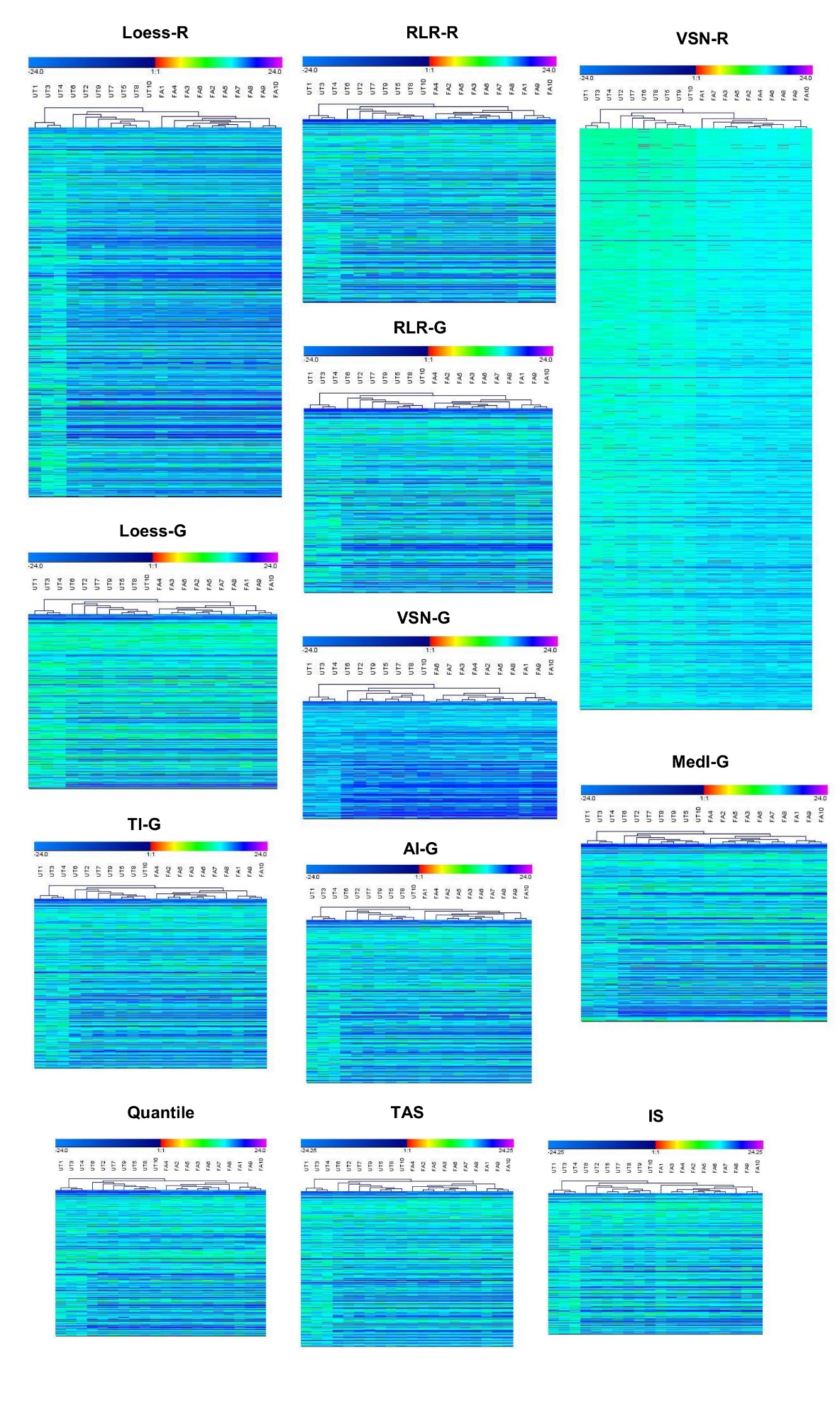


**Figure S15 – Dendrograms representing hierarchical clustering of control (UT) and test (FA) groups of dataset E based on differentiators identified using both p-value & fold change.**


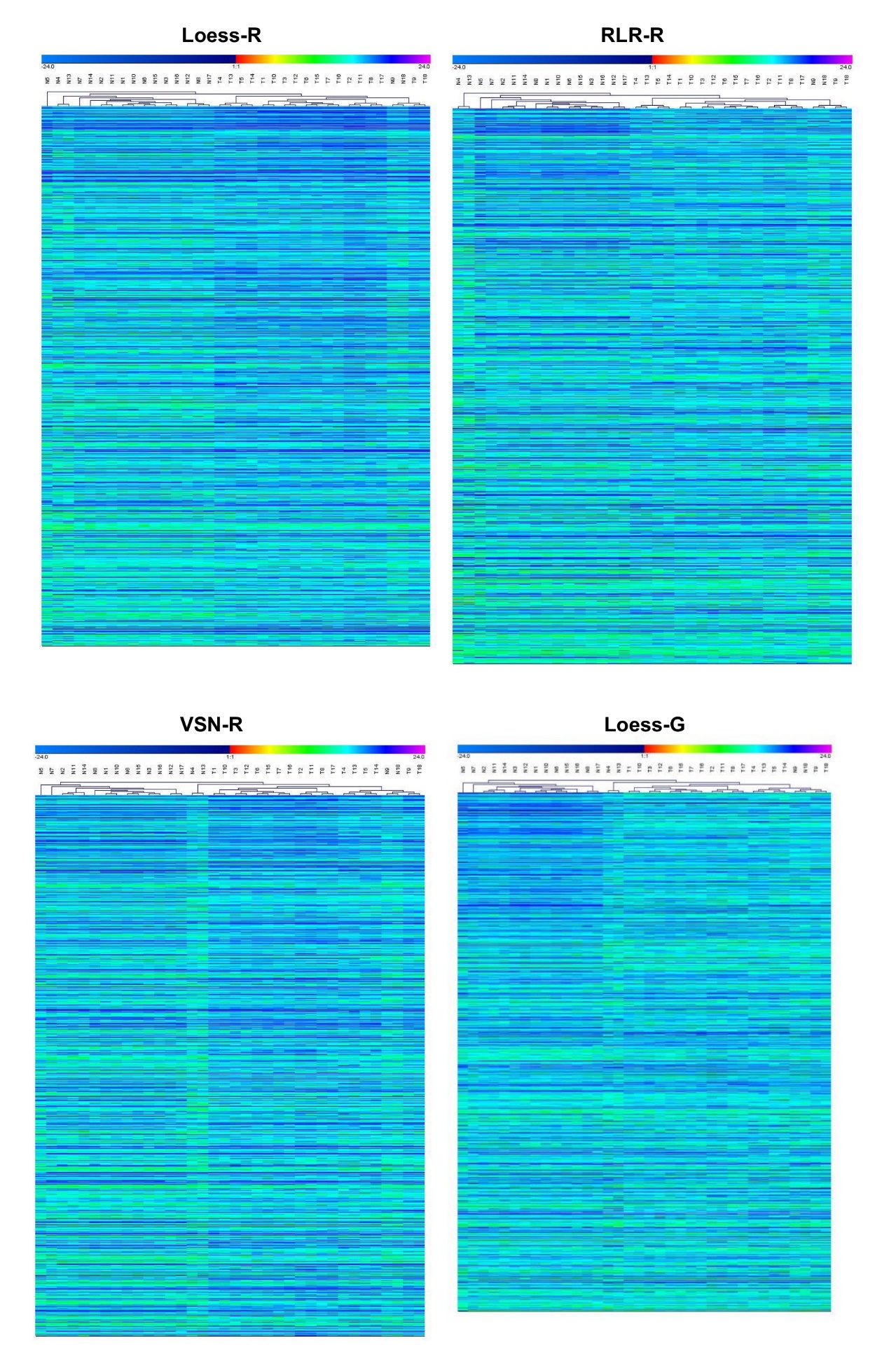


**Figure S16 – Dendrograms representing hierarchical clustering of control (N) and test (T) groups of dataset F based on differentiators identified using p-value.**


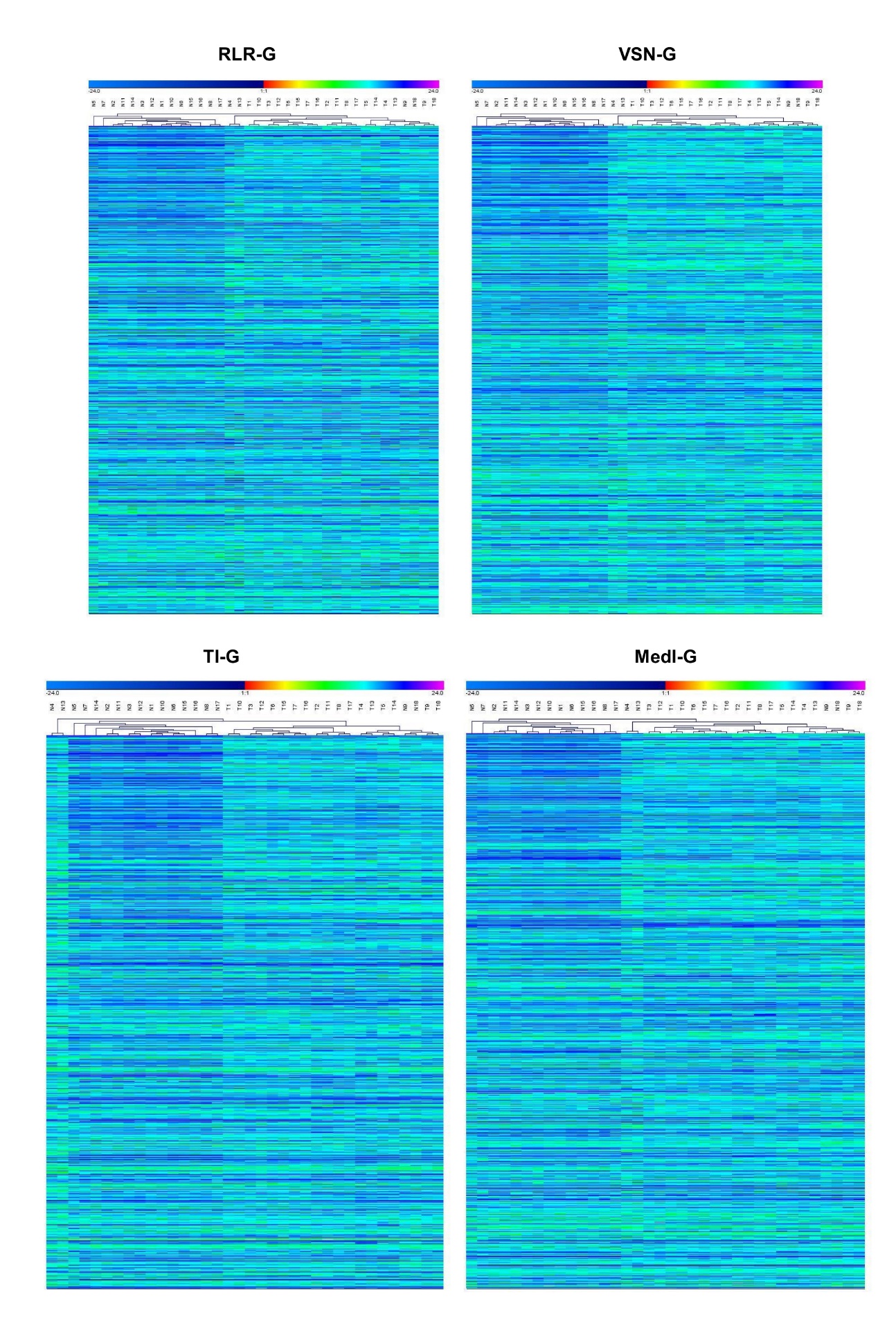


**Figure S16 (contd.) – Dendrograms representing hierarchical clustering of control (N) and test (T) groups of dataset F based on differentiators identified using p-value.**

**
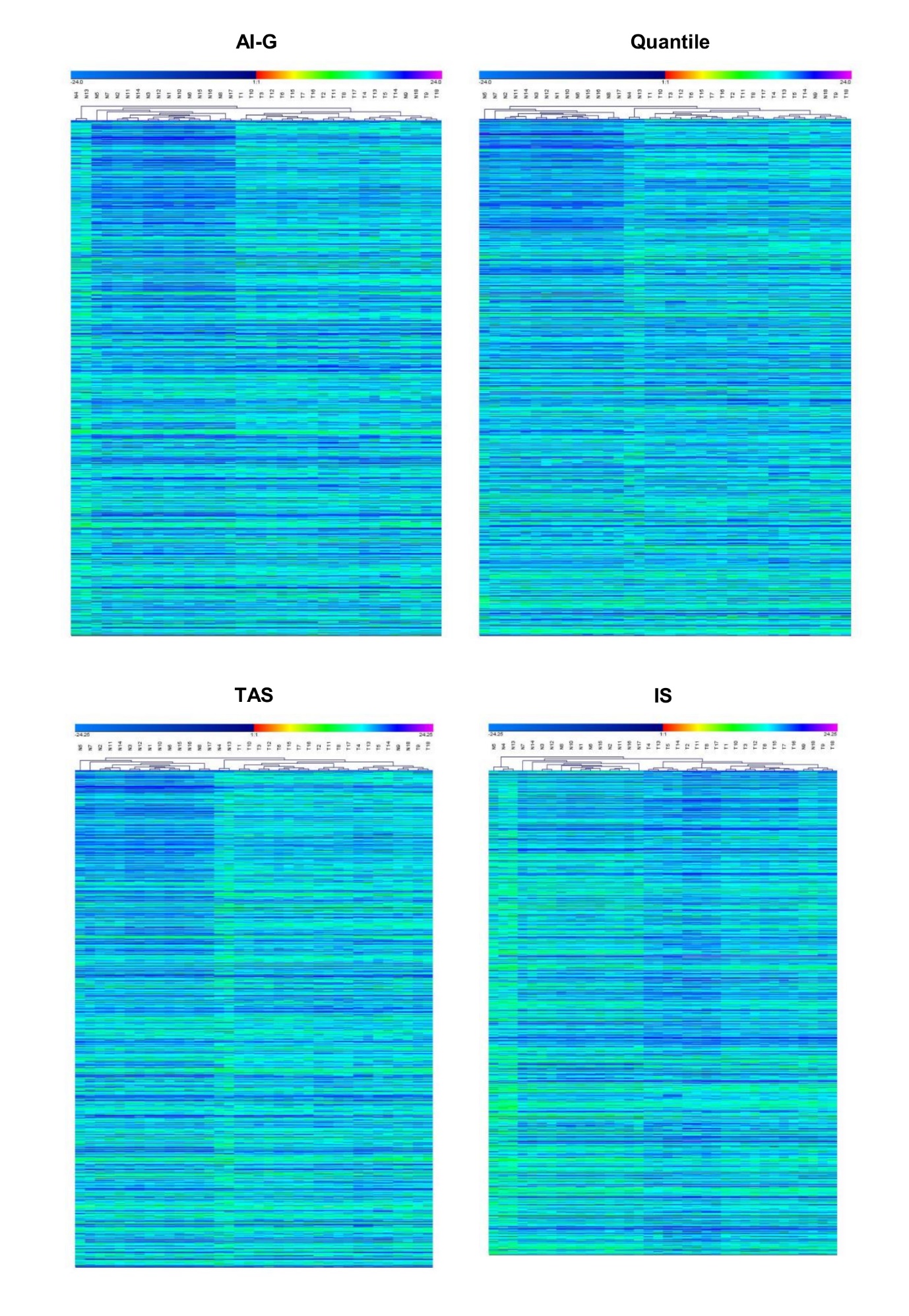
**

**Figure S16 (contd.)– Dendrograms representing hierarchical clustering of control (N) and test (T) groups of dataset F based on differentiators identified using p-value.**


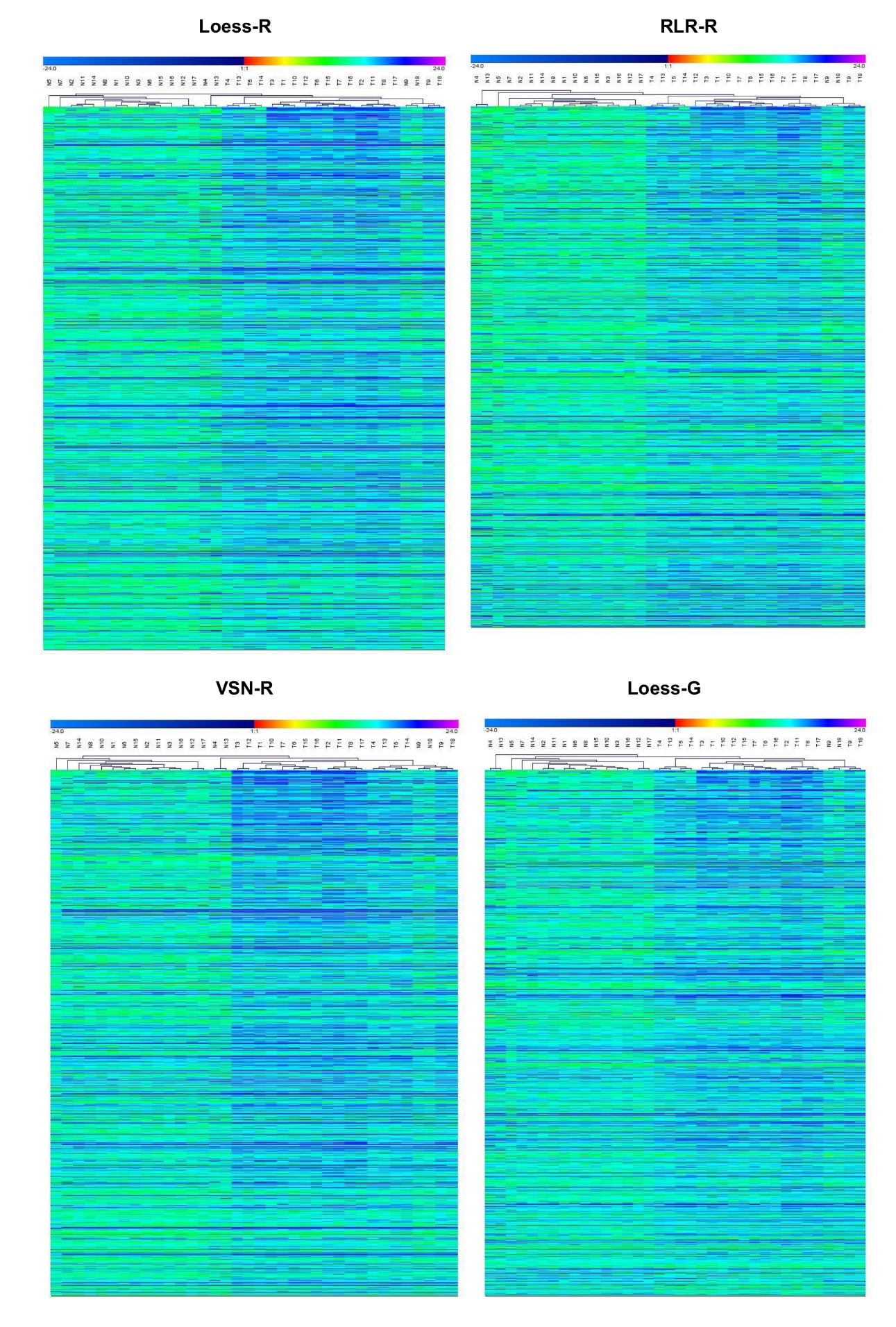


**Figure S17 – Dendrograms representing hierarchical clustering of control (N) and test (T) groups of dataset F based on differentiators identified using fold change.**


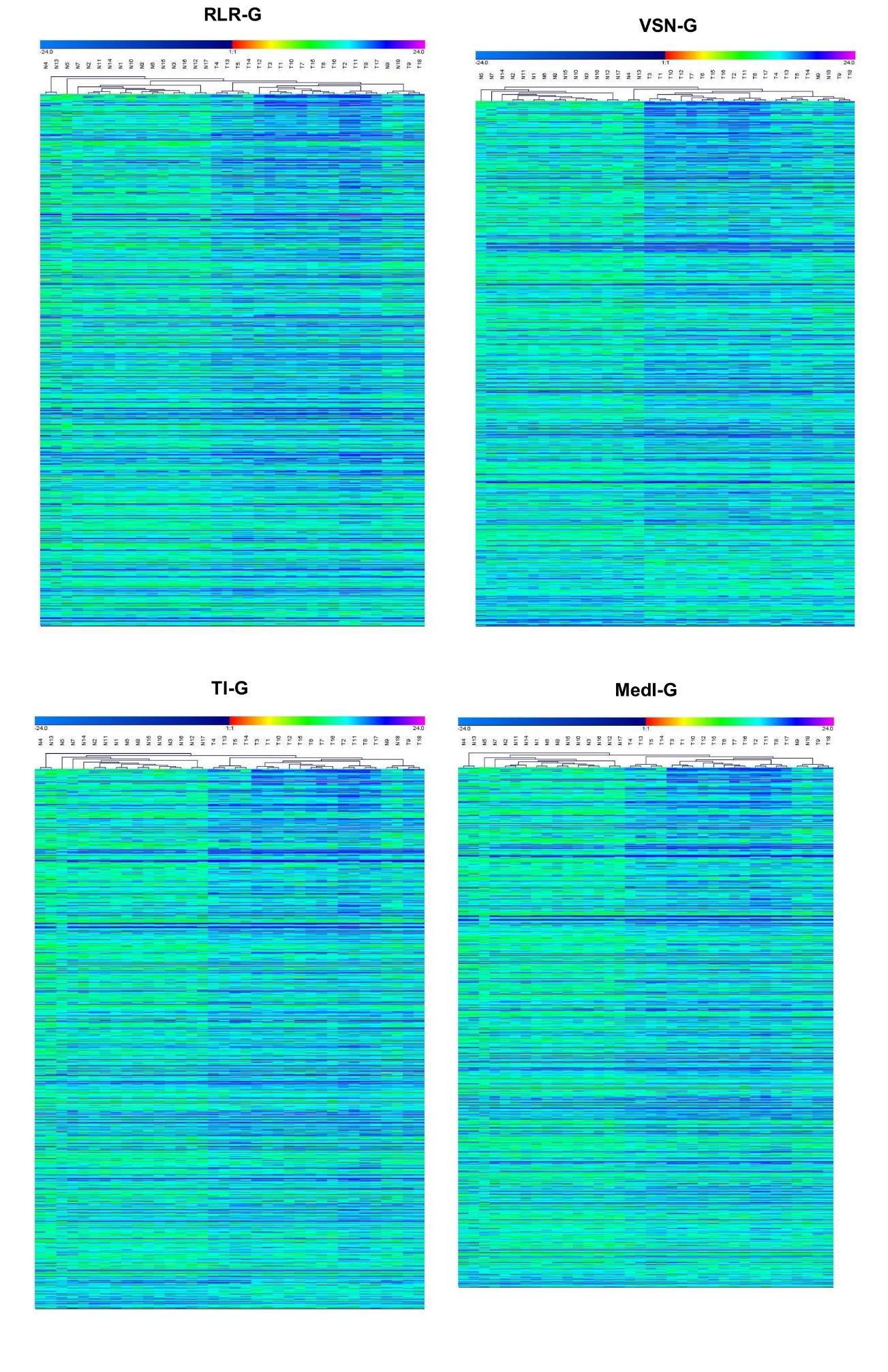


**Figure S17 (contd.)– Dendrograms representing hierarchical clustering of control (N) and test (T) groups of dataset F based on differentiators identified using fold change.**


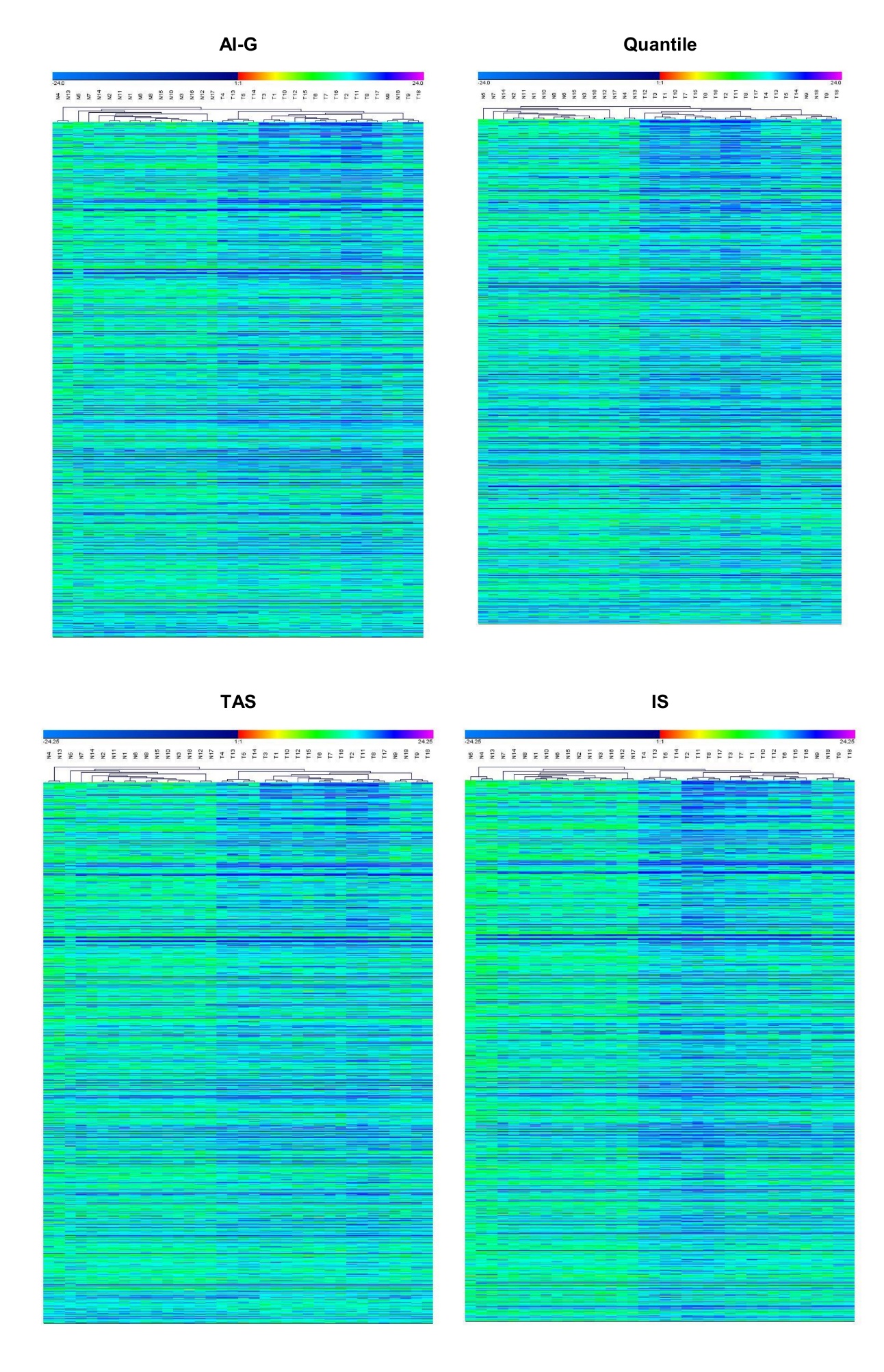


**Figure S17 (contd.)– Dendrograms representing hierarchical clustering of control (N) and test (T) groups of dataset F based on differentiators identified using fold change.**


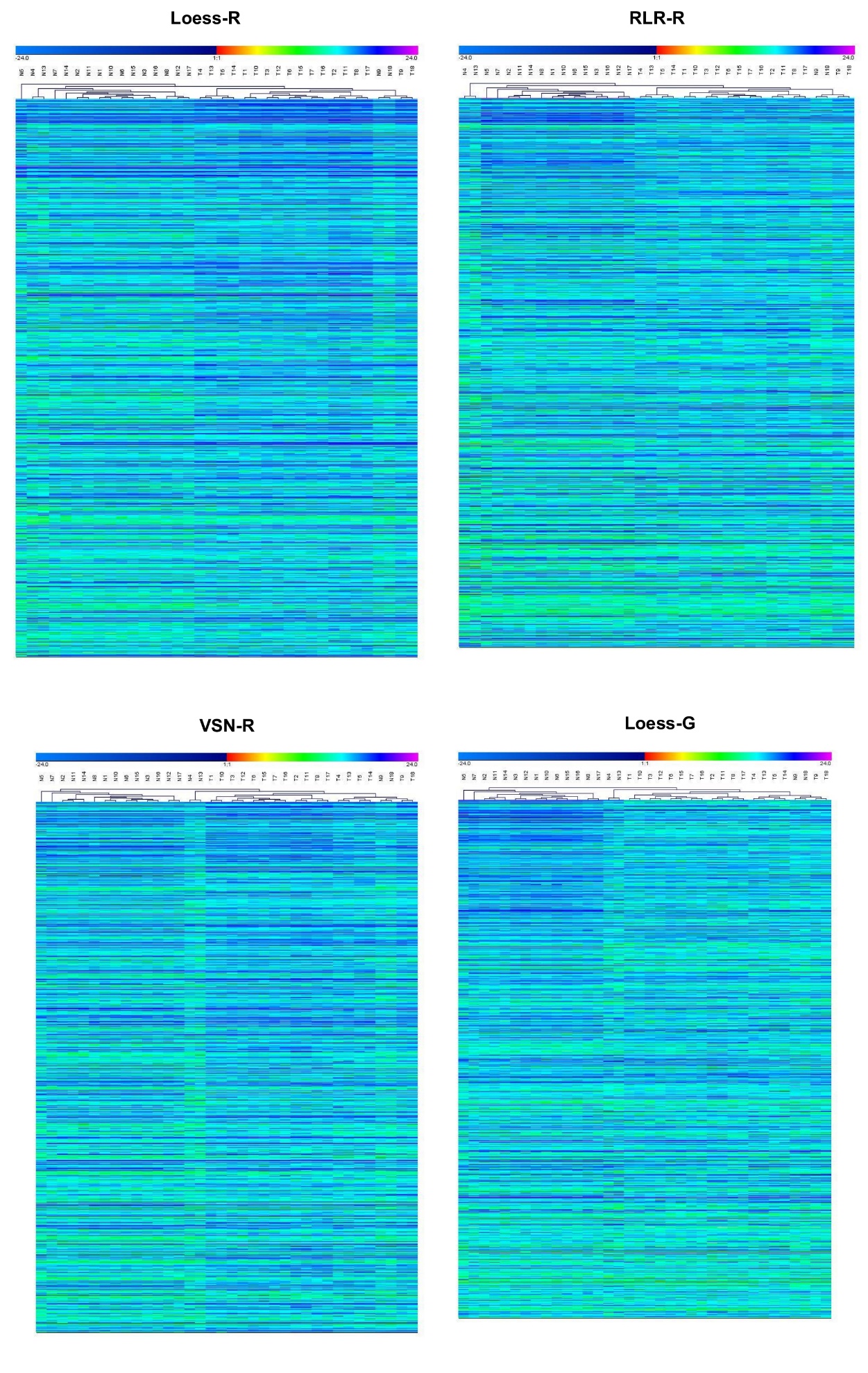


**Figure S18 – Dendrograms representing hierarchical clustering of control (N) and test (T) groups of dataset F based on differentiators identified using both p-value and fold change.**


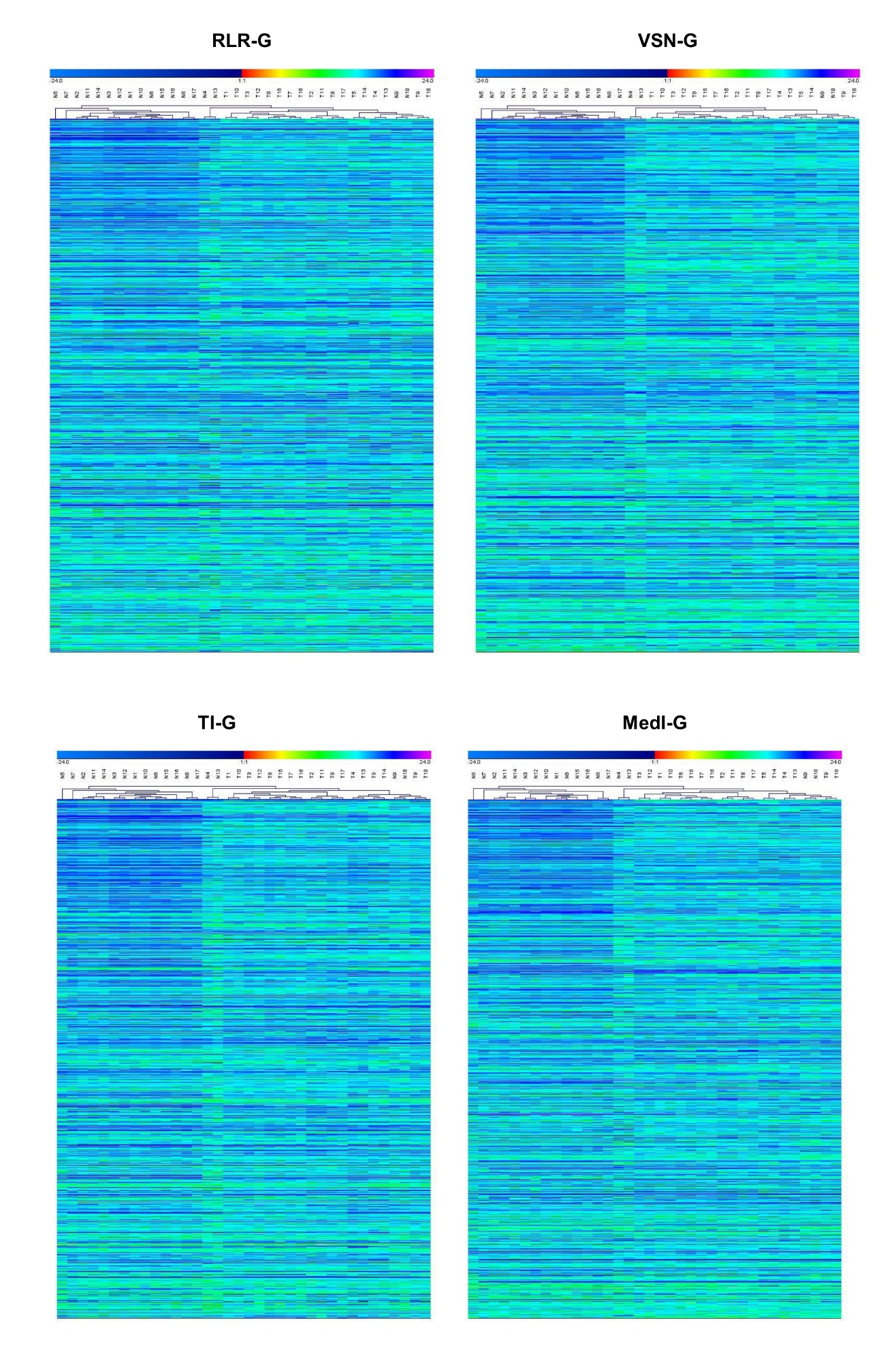


**Figure S18 (contd.) – Dendrograms representing hierarchical clustering of control (N) and test (T) groups of dataset F based on differentiators identified using both p-value and fold change.**


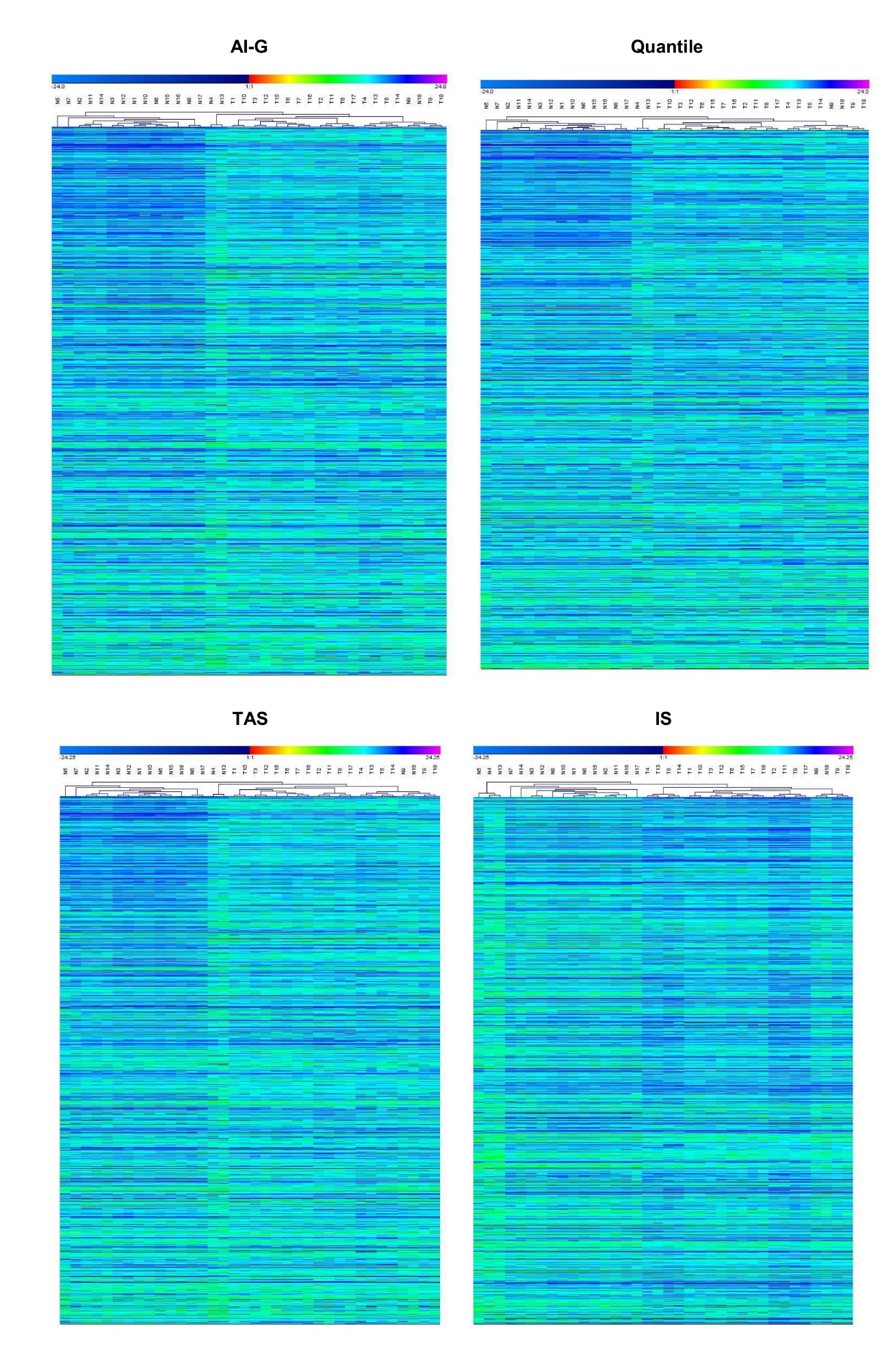


**Figure S18 (contd.)– Dendrograms representing hierarchical clustering of control (N) and test (T) groups of dataset F based on differentiators identified using both p-value and fold change.**
